# Distinct clonal lineages and within-host diversification shape invasive *Staphylococcus epidermidis* populations

**DOI:** 10.1371/journal.ppat.1009304

**Published:** 2021-02-05

**Authors:** Anna Both, Jiabin Huang, Minyue Qi, Christian Lausmann, Samira Weißelberg, Henning Büttner, Susanne Lezius, Antonio Virgilio Failla, Martin Christner, Marc Stegger, Thorsten Gehrke, Sharmin Baig, Mustafa Citak, Malik Alawi, Martin Aepfelbacher, Holger Rohde

**Affiliations:** 1 Institut für Medizinische Mikrobiologie, Virologie und Hygiene, Universitätsklinikum Hamburg-Eppendorf, Hamburg, Germany; 2 Bioinformatics Core, Universitätsklinikum Hamburg-Eppendorf, Hamburg, Germany; 3 Helios Endo-Klinik, Hamburg, Germany; 4 Institut für Medizinische Biometrie und Epidemiologie, Universitätsklinikum Hamburg-Eppendorf, Hamburg, Germany; 5 UKE Microscopy Imaging Facility, Universitätsklinikum Hamburg-Eppendorf, Hamburg, Germany; 6 Department of Bacteria, Parasites and Fungi, Statens Serum Institut, Copenhagen, Denmark; University of Tubingen, GERMANY

## Abstract

*S*. *epidermidis* is a substantial component of the human skin microbiota, but also one of the major causes of nosocomial infection in the context of implanted medical devices. We here aimed to advance the understanding of *S*. *epidermidis* genotypes and phenotypes conducive to infection establishment. Furthermore, we investigate the adaptation of individual clonal lines to the infection lifestyle based on the detailed analysis of individual *S*. *epidermidis* populations of 23 patients suffering from prosthetic joint infection. Analysis of invasive and colonizing *S*. *epidermidis* provided evidence that invasive *S*. *epidermidis* are characterized by infection-supporting phenotypes (e.g. increased biofilm formation, growth in nutrient poor media and antibiotic resistance), as well as specific genetic traits. The discriminating gene loci were almost exclusively assigned to the mobilome. Here, in addition to IS*256* and SCC*mec*, chromosomally integrated phages was identified for the first time. These phenotypic and genotypic features were more likely present in isolates belonging to sequence type (ST) 2. By comparing seven patient-matched nasal and invasive *S*. *epidermidis* isolates belonging to identical genetic lineages, infection-associated phenotypic and genotypic changes were documented. Besides increased biofilm production, the invasive isolates were characterized by better growth in nutrient-poor media and reduced hemolysis. By examining several colonies grown in parallel from each infection, evidence for genetic within-host population heterogeneity was obtained. Importantly, subpopulations carrying IS insertions in *agrC*, mutations in the acetate kinase (AckA) and deletions in the SCC*mec* element emerged in several infections. In summary, these results shed light on the multifactorial processes of infection adaptation and demonstrate how *S*. *epidermidis* is able to flexibly repurpose and edit factors important for colonization to facilitate survival in hostile infection environments.

## Author summary

*S*. *epidermidis* is a substantial component of the human skin microbiota, but also a major cause of nosocomial infections related to implanted medical devices. While phenotypic and genotypic determinants supporting invasion were identified, none appears to be necessary. By analysis of *S*. *epidermidis* from prosthetic joint infections, we here show that adaptive events are of importance during the transition from commensalism to infection. Adaptation to the infectious lifestyle is characterised by the development of intra-clonal heterogeneity, increased biofilm formation and enhanced growth in iron-free and nutrient-poor media, as well as reduced production of hemolysins. Importantly, during infection subpopulations emerge that carry mutations in a number of genes, most importantly the acetate kinase (*ackA*) and the β-subunit of the RNA polymerase (*rpoB*), have deleted larger chromosomal fragments (e.g. within the SCC*mec* element) or IS insertions in AgrC, a component of the master quorum sensing system in *S. epidermidis*. These results shed light on the multifactorial processes of infection adaptation and demonstrate how *S*. *epidermidis* is able to flexibly repurpose and edit factors important for colonization to facilitate survival under hostile infection conditions. While mobilome associated factors are important for *S*. *epidermidis* invasive potential, the species possesses a multi-layered and complex ability for adaptation to hostile environments, supporting the progression to chronic implant-associated infections.

## Introduction

S*taphylococcus epidermidis* is a major component of the human skin microbiota [1], but at the same time causes up to 30% of nosocomial bloodstream infections and over 30% of prosthetic joint infections (PJI), leading to high morbidity and gross excess health care costs[2–5]. For usually harmless commensal bacteria, infection represents an uncommon, likely disadvantageous event, and the term “accidental pathogen” has been coined to refer to this aspect [6]. *S*. *epidermidis* may not carry genomic resources directly dedicated to promote invasiveness, but it is very well able to re-purpose factors involved in colonization when infecting a host presenting with specific risk factors, most importantly implanted medical devices [7].

Understanding the transition routes from commensalism to invasive disease has been a main goal of *S*. *epidermidis* research, and work from the past decades has provided important insights into the molecular basis of device-related *S*. *epidermidis* infections [8–10]. Most importantly, biofilm formation has been recognized as a quintessential factor in the establishment of *S*. *epidermidis* infections [6,11,12].

Besides singular pathogenicity-associated factors, Multilocus Sequence Typing (MLST)-based analysis of population structure of *S*. *epidermidis* has found a significant over-representation of specific clonal *S*. *epidermidis* lineages in infections [13,14]. Sequence type 2 (ST2) is the most prominent lineage with worldwide dissemination and carries pathogenesis-relevant genotypic (e.g. *icaADBC*, *IS*256) and phenotypic (i.e. biofilm formation) traits. ST2 is especially well-adapted to the health care environment, harbouring a plethora of antimicrobial and antiseptic resistance genes [13,15,16]. Despite the over-representation of ST2 in infections, about 20–60% of *S*. *epidermidis* infection isolates appear to derive from diverse genetic backgrounds and pathogenic potential is clearly not restricted to single *S*. *epidermidis* lineages [13,17–19]. The success of *S*. *epidermidis* as a pathogen may therefore not strictly depend on a defined subset of genes or a specific genetic background, but rather on the ability to flexibly adapt to challenging environmental conditions after translocation from the colonizing habitat to the site of infection. The fact that *S*. *epidermidis* is able to colonize in a variety of conditions hints at its outstanding potential for adaptation which ultimately could benefit the pathogen in persisting in chronic infections [1]. The basis of such adaptive processes are dynamic tuneable, regulatory networks [8,20,21]. Previous *in vitro* studies in *S*. *epidermidis* have shown that it undergoes important changes in its expression profile and metabolic activity between its planktonic growth and biofilm states [22]. As an element of this transition, activity of the accessory quorum sensing regulator *agr* was shown to decrease, which in turn mediates an increase in biofilm formation and reduced production of cytotoxic molecules (phenol soluble modulins, PSMs) [22,23]. In the related species *Staphylococcus aureus* differentiation into quorum-sensing-mediated bi-stable phenotypes through positive feedback loops depending on environmental cues has been described [24].

Apart from regulatory events on transcription level, genetic studies have shown that within host evolution significantly contributes to pathogenesis of chronic bacterial infections [25]. On the human skin, *S*. *epidermidis* seems to evolve mainly through recombination and acquisition of mobile genetic elements [26]. More recently, reports indicate that *S*. *epidermidis* isolated over the time course of chronic infections potentially evolve infection relevant phenotypes (e.g. antibiotic tolerance, biofilm formation, small colony variants) through mutations at the single nucleotide level [9,27–29]. However, the extent and relevance of adaptation processes during the progression from commensalism to infection are still unclear today.

The overarching aim of this study is to make use of high resolution genomic and phenotypic analyses to gain comprehensive insights into potential adaptive events occurring during transition of *S*. *epidermidis* populations from commensalism to infection. To achieve this a prospective collection of nasal (i.e. commensal) and infection-associated strains derived from 23 patients suffering from PJI was established and subjected to extensive comparative, phenotypic and genomic analysis. In contrast to previous genomic studies which included *S*. *epidermidis* isolates from a great variety of infection types and mostly commensal control groups from healthy individuals, here invasive *S*. *epidermidis* isolates from a defined infection type were compared to commensal *S*. *epidermidis* isolates from the same patient. This patient-matched approach takes into consideration the host variation, thereby reducing the bias arising from differences between commensal community- and hospital associated *S*. *epidermidis* populations [18,30]. Importantly, this isolate collection enabled direct comparison of commensal and invasive representatives descending from one clonal lineage, allowing for analysis of within-clone adaptation to the infection environment. In the same line, population heterogeneity within the infection was analysed by sampling multiple colonies from each infection and important insights into genetic and phenotypic diversification were gained.

## Results

### Patient characteristics

Twenty-three patients with clinical signs of PJI were included into the study based on the recovery of *S*. *epidermidis* from at least one preoperatively collected joint fluid and from one intraoperative specimen. Thus, all patients fulfilled criteria of the Musculoskeletal Infection Society (MSIS) for the diagnosis of PJI [31]. Nasal swabs and intra-operative specimens (either joint fluid or tissue) were collected during revision surgery and sent to the study laboratory.

The patient collective consisted of eight females and 15 males, mean age was 69.4 years (range 52–84 years) (S1 Table, S2 Table). 18 patients had developed PJI after total hip arthroplasty and five patients after total knee arthroplasty. PJI occurred a median of 16 months after implantation of the prosthetic joint (range 1–182 months). Median duration of symptoms as reported by the patient (pain, inability to walk, instability) was 6 months (range 1–46 months). Patients did not receive antibiotic therapy in at least two months prior to revision surgery.

### Isolate collection

Usually, *S*. *epidermidis* occurs as a harmless component of human skin microbiota with a low pathogenic potential. We therefore investigated whether invasive *S*. *epidermidis* strains exhibit systematic phenotypic or genotypic differences in comparison to commensal strains. Two to twelve colonies (median = 10) grown from an intra-operative infection specimen were picked. Pulsed-field gel electrophoresis (PFGE) was performed on all isolates. All colonies from each respective infection (infection isolates, referred to as INF isolates, total of analysed infections, n = 23) were PFGE-identical i.e. clonally identical.

In order to establish a rigorous control group for the PJI-derived INF isolates, a nasal swab was plated and 14–28 commensal *S*. *epidermidis* isolates were picked and subjected to PFGE. Zero to six isolates (median = 3) with individual PFGE-patterns, different from the INF strain could be identified per patient (non-clonal nose isolates, referred to as nCloNo isolates, n = 62) (S3 Table).

**Fig 1:**
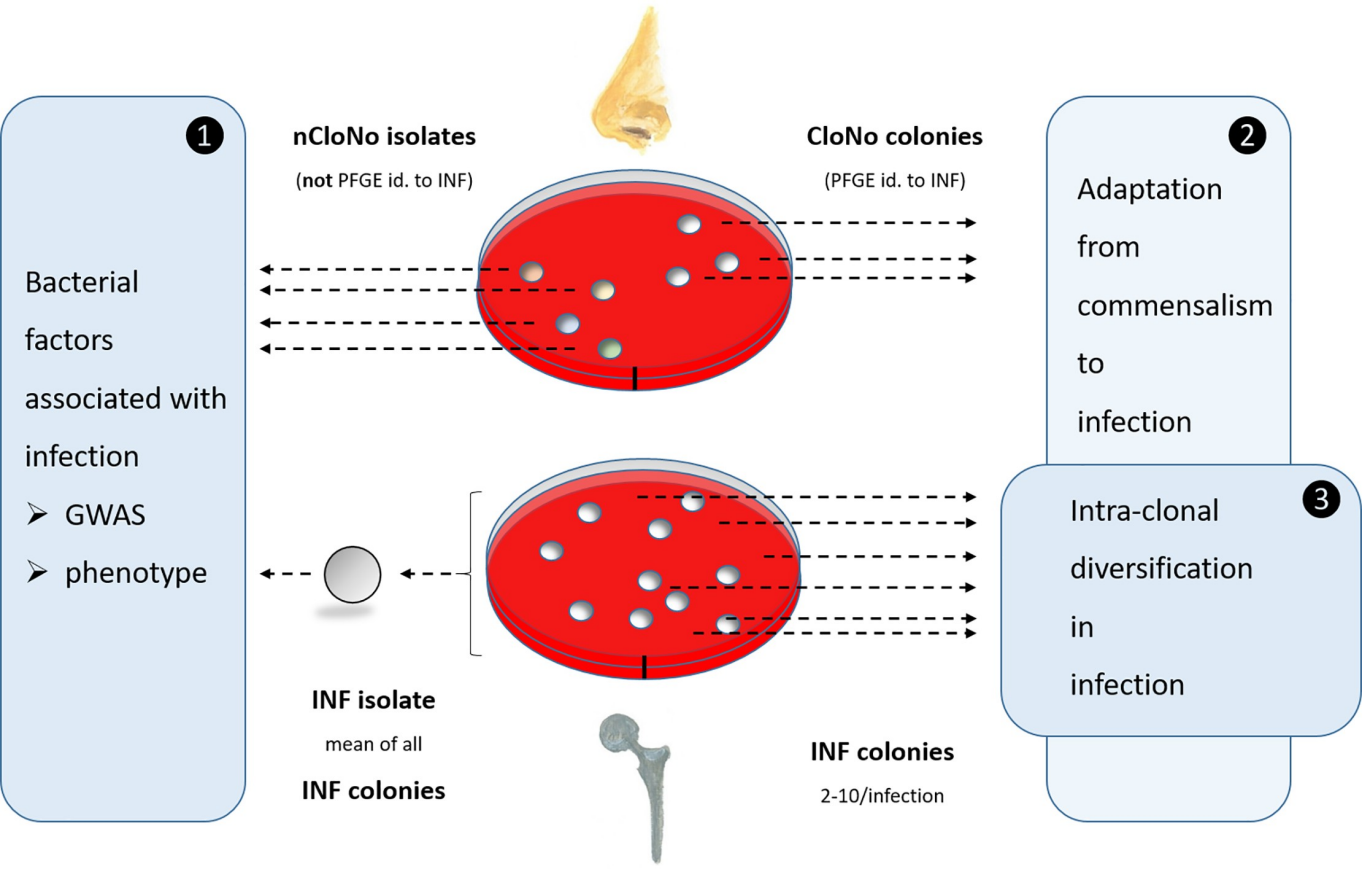
Overview of sampling procedure at the example of one individual patient. **(1)** For comparison of phenotypes and genotypes of infection and commensal isolates. Phenotypic values for INF isolates are aggregated measured values of all picked colonies from each infection, thus resulting in one representative INF isolate per infection. These INF isolates (n = 23) were compared to nCloNo isolates to identify systematic differences between infection-associated and commensal *S*. *epidermidis* isolates. **(2)** INF colonies were compared to CloNo colonies (PFGE-identical to the same patient’s INF colonies) in order to identify differences in phenotype and genotype between PFGE-identical isolates derived from commensal and infection niches. **(3)** All INF colonies (n = 2–12 per infection) from each infection were analysed for phenotypic and genotypic diversification within the infection.

Besides those clonally diverse commensal strains, in seven of 23 patients, PFGE of nasal *S*. *epidermidis* isolates yielded patterns identical to the corresponding INF isolate) (S4 Table), thus likely descendants of the commensal precursors of the INF isolate (clonal nose isolates, referred to as CloNo isolates). These were only used to study parallel evolution in the commensal niche in relation to evolution of the corresponding INF isolates in the joint (Fig 1).

### Phenotypic comparison of invasive and non-invasive *S*. *epidermidis*

#### *S. epidermidis* strains from PJI display phenotypes that facilitate persistence under stress conditions

In order to compare phenotypes of INF isolates with commensal nCloNo isolates, several phenotypes relevant to infection establishment were measured. Values for INF isolates are aggregated measured values of all picked colonies from each infection.

**Fig 2:**
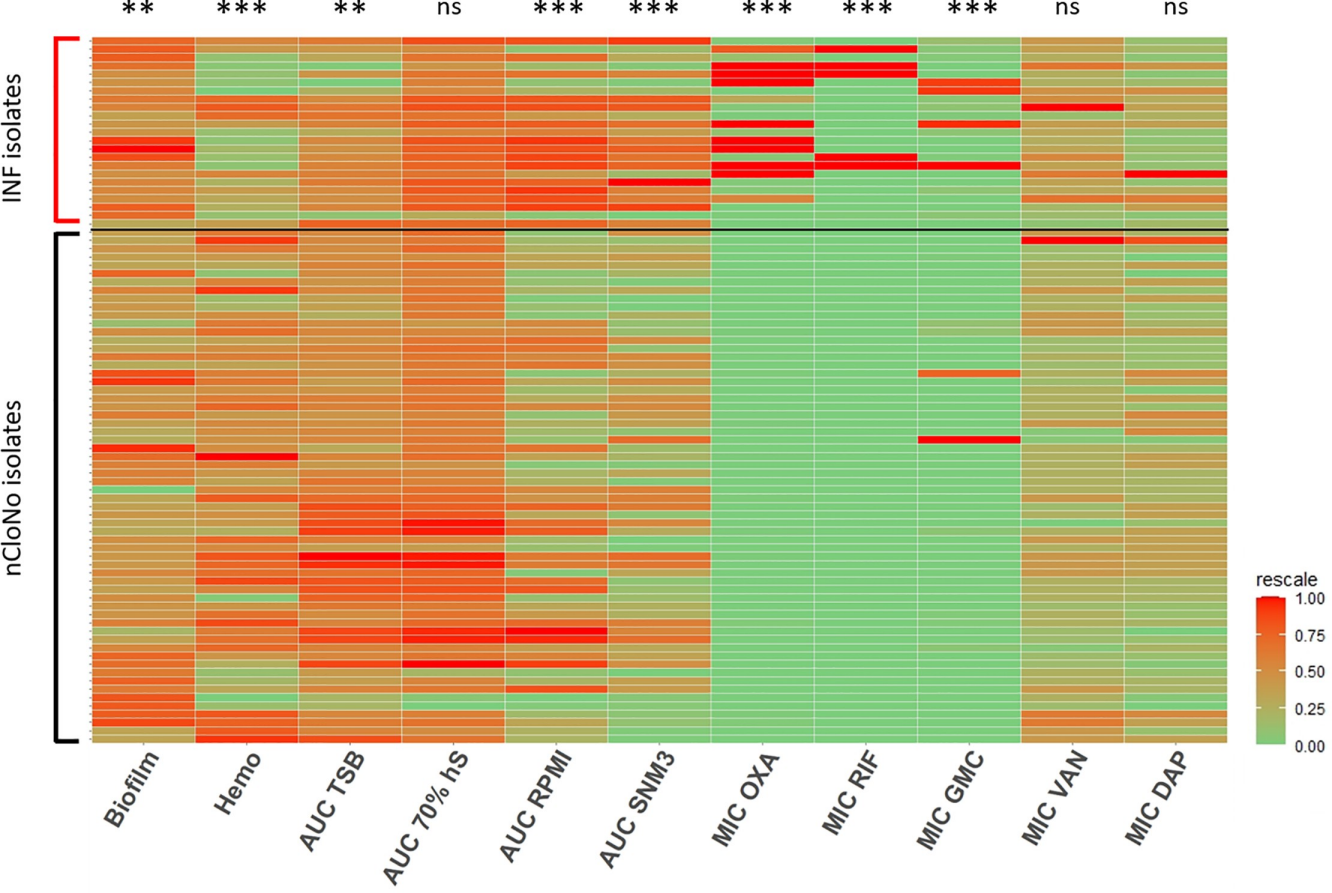
Phenotypes of INF isolates and nCloNo isolates. Heat map of all continuous phenotypes tested in this study. Values for INF isolates are aggregated measured values of all picked colonies from each infection. Phenotypes in columns are rescaled by column. Each row represents one isolate. Columns represent phenotypes: Biofilm formation in TSB (log_10_-transformed), hemolysis (Hemo, extinction of free haemoglobin after incubation of 2% sheep erythrocytes with overnight culture supernatants), growth curve AUCs in TSB (AUC TSB, 22h static incubation, OD_600_ measurement every 30min), growth curve AUCs 70% human serum in PBS (AUC 70% hS, same conditions, log_10_-transformed), growth curve AUCs RPMI (AUC RPMI, log_10_-transformed), growth curve AUCs in synthetic nose media (AUC SNM3), minimal inhibitory concentrations (MIC) of oxacillin (MIC OXA), rifampicin (MIC RIF), gentamicin (MIC GMC), vancomycin (MIC VAN) and daptomycin (MIC DAP) Levels of significance indicated by *(p≤0.05), **(p≤0.01), *** (p≤0.001). Significance testing by Student’s *t*-test for biofilm formation hemolysis and growth (areas under the curve, AUC), significance testing of susceptibility testing data by categories (susceptible vs. resistant) by Pearson’s Chi-square.

Biofilm formation facilitates *S*. *epidermidis* persistence by providing protection from host immune responses. In line with this, INF isolates produced two-fold stronger biofilms in tryptic soy broth (TSB) compared to nCloNo isolates (p = 0.004, 95%-CI: 1.2–3.5) (Fig 2 and S1 Fig), as determined by microtiter plate assay of mature biofilms.

During *S*. *epidermidis* infections, cytolytic phenol soluble modulin (PSM) peptides are essential triggers for neutrophil proinflammatory responses, while simultaneously modifying *S*. *epidermidis* biofilm formation[32]. Quantification of sheep erythrocytes hemolysis was therefore used to quantify PSM production in INF and nCloNo isolates[33]. In INF isolates, hemolysis was reduced by 0.7-fold (p<0.001, 95%-CI: 0.6–0.9), indicating a low inflammatory, biofilm-supporting phenotype. On the other hand, proteolysis of skim milk proteins, as surrogate for secreted protease production, was more often observed in INF isolates (72.7%) compared to nCloNo isolates (46.9%), but the difference was not significant (p = 0.08).

Bacterial growth was tested in optimal nutrient rich media (TSB) and nutrient-restricted media, namely RPMI-1640 for cell culture (low in carbon-sources, iron-free), 70% [vol/vol] pooled heat-inactivated human serum and synthetic nose media (SNM3, containing amino acids, vitamins and ions, as found in the human nose, low in carbon sources, iron free[34]). The area under the growth curve (AUC) was used for quantification. Interestingly, nCloNo isolates were growing slightly better in nutrient-rich TSB compared to INF isolates (AUC 1.2-fold higher, p = 0.008). However, in iron-free and nutrient-poor RPMI and SNM3, INF isolates showed increased growth compared to nCloNo isolates (RPMI AUC 2-fold higher, p = 0.001 and SNM3 AUC 1.8-fold higher, p<0.001, respectively). There was no significant difference in growth in 70% human serum (Figs 2 and S1 Fig). Taken together, these data indicate that compared to patient-matched, colonizing *S*. *epidermidis* isolates, invasive *S*. *epidermidis* isolates are equipped with phenotypic traits that facilitate persistence in hostile environments as encountered in PJI.

#### *S. epidermidis* strains from PJI are significantly more resistant to antibiotics than commensal strains

Resistance to oxacillin, gentamicin, rifampicin, quinolones, cotrimoxazol and fosfomycin was significantly more common in *S*. *epidermidis* INF isolates compared to nCloNo isolates (S5 Table). Vancomycin susceptibility was retained in all isolates. Resistance against β-lactams, gentamicin and quinolones was highly associated with hospital-associated MLSTs ST2 and ST5 (p<0.001). Antibiotic resistance rates were similar in ST2 and ST5, only resistance to rifampicin (33% vs. 0%, respectively, p = 0.038) and cotrimoxazol (55% vs. 0%, respectively, p = 0.004) was more common in ST2.

### Genotypic comparison of invasive and non-invasive *S*. *epidermidis* isolates

#### Distinct genetic backgrounds facilitate infection establishment in *S. epidermidis*

**Fig 3:**
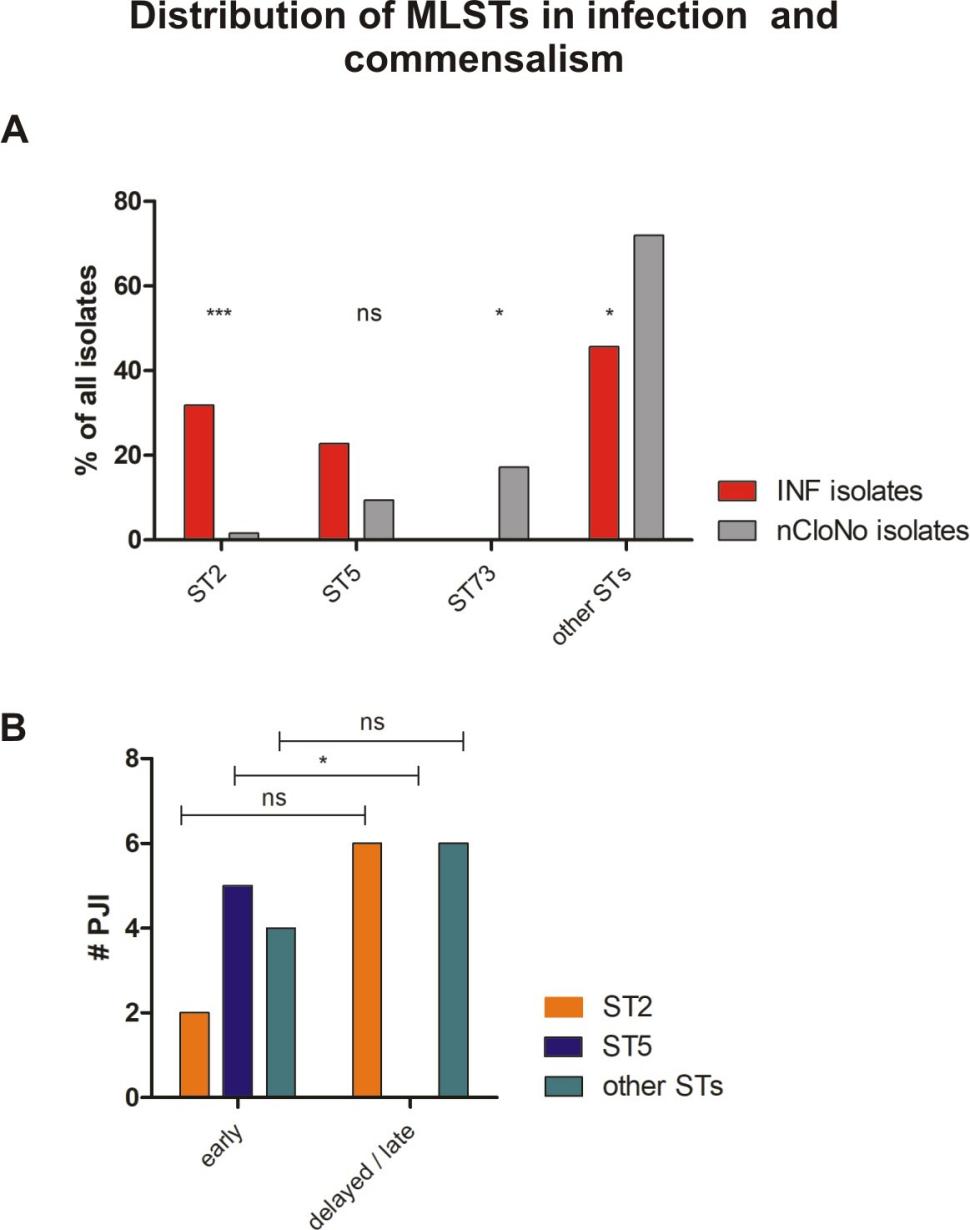
**(A) Distribution of Multilocus Sequence Types (MLSTs) in INF isolates and nCloNo isolates.** Red bars (INF isolates) and grey bars (nCloNo isolates) represent the proportion of indicated sequence type in invasive and commensal isolates, respectively. **(B)** Distribution of STs in early and delayed/late PJI. ST2 is represented by an orange, ST5 by a blue and all other STs by a green bar. Early infections: occurrence within 0–3 months after surgery, delayed/late infections: occurrence >3 months after surgery. Levels of significance indicated by *(p≤0.05), **(p≤0.01), ***(p≤0.001).

Building on the finding that infection-associated *S*. *epidermidis* isolates exhibit phenotypes facilitating persistence, the hypothesis was pursued that infection is linked to specific genotypes, which further the pathogen’s ability to establish and maintain infections. Multi locus sequence typing (MLST), based on sequence comparison of seven core genome loci, is less discriminatory compared to PFGE, but allows for classification into global lineages [35]. MLST of all isolates showed that nCloNo isolates were highly diverse (genotypic diversity index h = 0.95). Similarly, INF isolates also derived from a variety of sequence types (Fig 3A and S2 Fig), but were overall less diverse (h = 0.81)[36]. In particular, 8/23 INF isolates belonged to ST2, while only 1/62 nCloNo isolates belonged to that particular sequence type (p<0.001). By phylogenetic analysis, all ST2 isolates belonged to a multidrug-resistant lineage of global spread, previously designated “ST2-mixed” [15] (S3 Fig).

**Fig 4:**
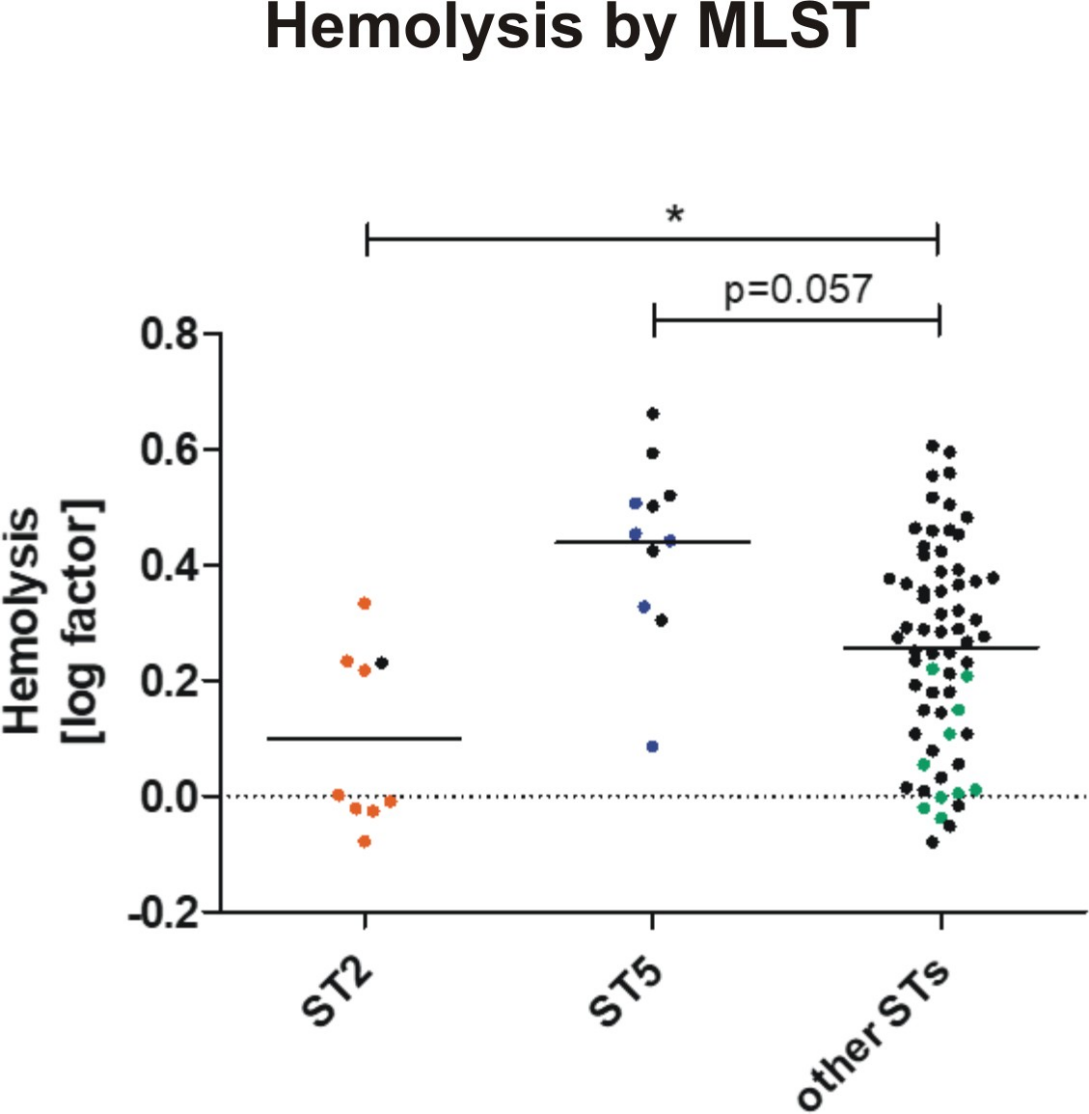
Hemolytic activity in different *S*. *epidermidis* STs. Overnight culture supernatants from ST2 (INF isolates in orange, n = 8. nCloNo isolates in black, n = 1), ST5 (INF isolates in blue, n = 5. nCloNo isolates in black, n = 6) and all other STs (INF isolates in green, n = 10. nCloNo isolates in black, n = 56) were investigated for hemolytic activity (lysis of sheep erythrocytes [2%]), and results were analysed with Student’s *t-*test. For INF isolates aggregated values of all colonies per one infection were used.

Importantly, in early PJI (≤3 months post-implantation) only a minority of infection strains belonged to ST2 (2/11), while in delayed and late PJI (>3–12 months and >12 months[37]), ST2 constituted half of all detected MLSTs (6/12, p = 0.11). In contrast, ST5 was found in 5/11 early infections, but not in later infections (p = 0.013)(Fig 3B). It is generally hypothesized that *S*. *epidermidis* PJI mostly occur through inoculation of the joint at the time of implantation. Possibly, ST5 might cause earlier onset of symptoms, due to a more aggressive phenotype. Indeed, isolates of ST5 were significantly more active in hemolysis compared to all other strains (p = 0.002), while ST2 showed a trend towards less hemolysis (p = 0.057)(Fig 4). Concerning mobile genetic elements, Staphylococcal Cassette Chromosome *mec* (SCC*mec*) elements, previously associated with infection, were more common in INF isolates (17/23 INF, 11/62 nCloNo isolates, p<0.001). Arginine catabolic mobile elements (ACME) were more commonly found in nCloNo isolates (8/23 INF, 41/62 nCloNo, p = 0.009), and have previously been associated with commensalism (S4 Table)[38,39].

#### Agr-typing

The *agr* operon (*agrBDCA*) encodes a quorum sensing system, conserved in many staphylococcal species. Briefly, it codes for an precursor auto-inducing peptide (AgrD) which is modified and exported by AgrB and sensed by a two-component sensor signal transduction system (histidine kinase sensor AgrC and response regulator AgrA) [40]. It plays a role in the regulation of biofilm formation, PSM, δ-Toxin and exoprotease production in *S*. *epidermidis* [41,42].

The Agr quorum sensing system in *S*. *epidermidis* is divided into three groups based on the aminoacid sequence of AgrD. In our study population, Agr type 1 was the most common form in both INF and nCloNo isolates (13/23 and 30/62, respectively, difference not significant). Agr type 2 was found in 10/23 INF isolates, while it was rarer in the nCloNo group (11/62 nCloNo isolates, p = 0.024). Interestingly, Agr type 3 was only found in nCloNo isolates (20/62, p = 0.001). In our strain collection Agr types correlated strictly with MLST. For example ST2 made up the majority of Agr type 1 INF strains, while ST5 was major in the Agr 2 group. The Agr type 3 group was dominated by isolates belonging to ST73 or ST218 (S4 Table).

Loss of Agr function has previously been described as a contributing factor in infections caused by *Staphylococcus sp*.. It appears to contribute towards a phenotype furthering immune evasion, such as increased biofilm formation and reduced production of PSMs [43–46]. We did not observe any non-synonymous SNPs (nsSNPs) or small insertions or deletions in *agrBDCA*. However, there were colonies carrying insertion sequences (IS*256* and IS*1182-*family*)* in *agrC* in six infections (see below). This was not observed in nCloNo isolates.

#### Mobile genetic elements are associated with invasiveness in *S. epidermidis*

**Fig 5:**
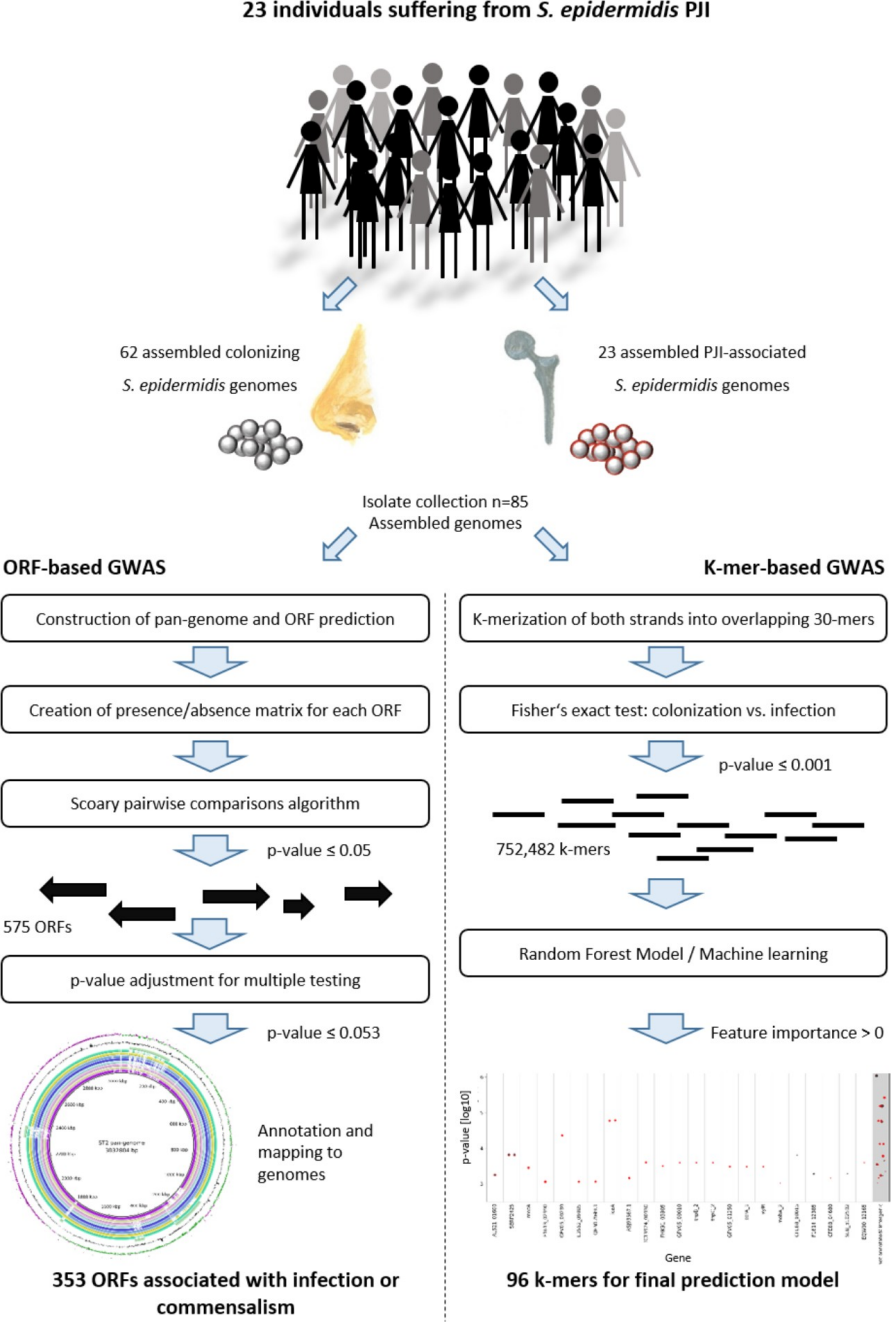
Overview of ORF-based and k-mer-based genome-wide association study of colonizing and PJI-associated *S*. *epidermidis* isolates. GenBank accession numbers for all isolates, S3 Table.

The species of *S*. *epidermidis* carries a massively diverse accessory genome, with only about 28% of accessory genes functionally annotated [38]. The fact that some sequence types were more commonly isolated from infection, led to the hypothesis that these STs may be associated with accessory genetic traits improving their ability to establish and maintain infection. To test this idea, an Open reading frames (ORF)-based genome-wide association study (ORF-based GWAS) was conducted to identify loci from the accessory genome associated with infection (Fig 5). ORFs were predicted from the pan-genome of all *S*. *epidermidis* isolates sequenced for this study and presence or absence was called for each isolate (INF isolates, n = 23, nCloNo isolates, n = 62) with a tolerance of 95% sequence similarity. 575 genes had a native p-value ≤0.05, p-values were then adjusted for multiple testing, so that 353 genes with an adjusted p-value ≤0.052 remained (299 infection associated, 53 commensalism associated). Importantly, 93.8% of associated genes belonged to the mobilome, as defined by being phage-associated, plasmid-associated or mobile genetic elements (e.g. *IS*-elements, SCC*mec* or ACME)(Table 1).

**Table 1:** Overview of results from ORF-based gene-association study.

		# genes	OR^a^	adjusted p-value
**Infection-associated ORF**				
	**Mobilome (all)**	286		
	ΦSepi-HH1	44	11–18	0.01–0.052
	Phage-related island PI-Sepi-HH2	45	6-∞	0.002–0.035
	ΦSPbeta-like	139	11	0.052
	SCC*mec*	28	5–21	0.011–0.052
	partial SeRI*fusB*^b^	7	16-∞	0.007–0.02
	Plasmid encoded orfs IRL01 plasmid pBPH0747-01-like	14	8–26	0.004–0.04
	other phage associated	3		
	IS*256*		21.3	<0.001
	AAC(6′)-APH(2")		14.5	0.002
	*qacA*		8.9	0.008
	**Chromosome-encoded ORF (non-mobilome)**	14		
**Commensalism-associated loci**					
	**Mobilome (all)**	44		
	plasmid-encoded ORFs	25	0–0.1	0.001–0.052
	ACME	15	0.1–0.2	0.007–0.052
	**Chromosome encoded ORF (non-mobilome)**	9		
	*agrB* and *agrD* Type III	2	0	0.027

a Odds ratio for infection. Ranges of ORs and p-values include all loci within the named structure which occurred at variable frequencies.

^b^Partial SeRIfusB included seven genes (NCTC13924_01702, NCTC13924_01705..01710), which are most likely associated to a phage-related resistance islands.

The bacterial insertion element *IS*256 was the most significant infection associated locus (OR = 21.3, adj. p<0.001), followed by the aminoglycoside actyltransferase AAC(6′)-APH(2")(OR = 14.5, adj. p = 0.002). The previously described association of SCC*mec* elements with invasive *S*. *epidermidis* isolates was confirmed, with 28 genes related to the various variants of the element being significantly associated [38].

**Fig 6:**
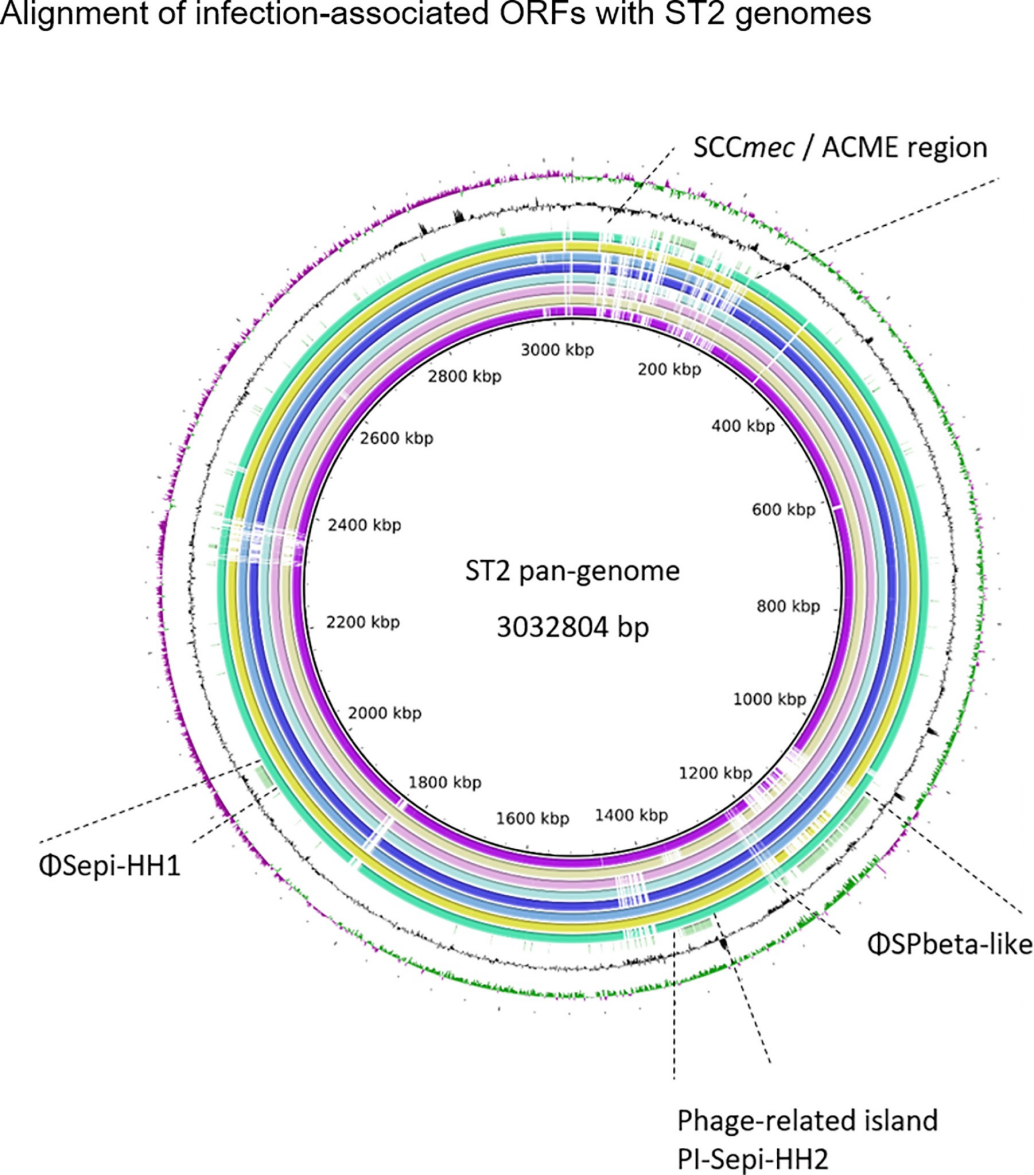
Alignment of infection-associated genes against ST2 pan-genome. From innermost circle to outermost: HD05 (purple), HD12 (lime), HD21 (pink), HD25 (light blue), HD31 (dark blue), HD46 (blue), HD47 (yellow), HD99 (turquois) and significantly associated genes (green). GC-content (black), GC-skew (+) green, GC-skew (-) purple.

Surprisingly, 226/299 infection associated genes mapped to three prophage regions (Fig 6 and S4 Fig). While one was found to be similar to previously described staphylococcus phage SPbeta-like staphylococcus phage (NC_029119.1, 86% sequence identity)[47] and will here be referred to as ΦSPbeta-like, (GenBank accession number MT880872) the remaining two regions did not correspond to any previously described *Staphylococcus* phage[48] and will here be referred to as ΦSepi-HH1 (MT880870) and phage-related island PI-Sepi-HH2 (MT880871). Both regions contained structural phage genes which were assigned with high probability to the order of *Caudovirales*. As by phaster analysis [49], prophage ΦSepi-HH1 was complete, while PI-Sepi-HH2 was annotated as incomplete. ΦSepi-HH1 was present in all analysed ST2 (n = 9), as well as in all isolates of ST83 (9/23 INF isolates, 3/62 nCloNo isolates). PI-Sepi-HH2 was not present in all ST2 isolates (8/9), but large fragments of the prophage were also found in isolates belonging to ST290, ST297 and ST487 (9/23 INF isolates, 6/62 nCloNo isolates). ΦSPbeta-like was found in 6/9 ST2 isolates, as well as in the only ST22 isolate from this study (5/23 INF isolates, 2/62 nCloNo isolates). Insertion sites were identical in all isolates.

Fourteen chromosomal loci, which could not be associated to any known mobile genetic elements, were associated with infection. One of these loci was annotated as AAC(6′)-APH(2") aminoglycoside transferase, for the other 13 loci no annotation or only very basic function prediction was available in the database (ncbi). This lack of annotation underlines the limited understanding of the content and function of the *S*. *epidermidis* accessory genome which we have so far.

Interestingly, the *icaADBC* locus, which has previously been found to be significantly over-represented in invasive isolates, was initially associated with infection in this isolate collection, but no longer was after adjusting for multiple testing (12/23 INF isolates, 15/62 nCloNo isolates, p = 0.02; adj. p = 0.16)[50,51].

Among the 53 commensalism associated genes, 15 were related to ACME, 25 were plasmid-encoded, two were associated to other mobile genetic elements and two were phage-associated. Nine loci were distributed over the chromosome, among these master quorum sensing system components *agrB* and *agrD* of type III, which were present in 20/62 commensal isolates, and absent from all infection isolates (OR = 0, adj. p = 0.027). The remaining seven were so far only annotated with a general function or domain prediction (S6 Table).

In addition to the ORF-based association study, we performed a k-mer based genome-wide association study (k-mer GWAS)(Fig 5). In contrast to the ORF-based GWAS, this approach allows the identification of discrete sequence differences in coding and non-coding regions, which may be the signature of specific STs or may influence the activity of the gene product. The assemblies of all sequenced isolates from this study was split into overlapping 30-mers. The association of each k-mer to infection and commensalism was tested with Fisher’s exact test. More than 750,000 k-mers were significantly associated (native p ≤ 0.001). A machine learning approach with the goal of establishing a prediction model based on a limited set of k-mers was employed (Fig 5 for details). 96 k-mers were included in the final prediction model (S6 Table). They mapped to a total of 55 genes, all part of the accessory genome, while 13 k-mers mapped to intergenic regions. Similarly to previous studies, eight of the infection-associated k-mers mapped to the SCC*mec*, two mapped to ACME and ten mapped to other mobile genetic elements. Interestingly, carbamate kinase *arcC* (12/96 k-mers), of the constitutive arginine catabolism gene cluster, and phosphonates transport permease *phnE* (6/96 k-mers) were overrepresented in the model, with some k-mers associated with infection and some with commensalism. Thus, allele variants in these two genes may have discriminatory power (S5 Fig).

**Fig 7:**
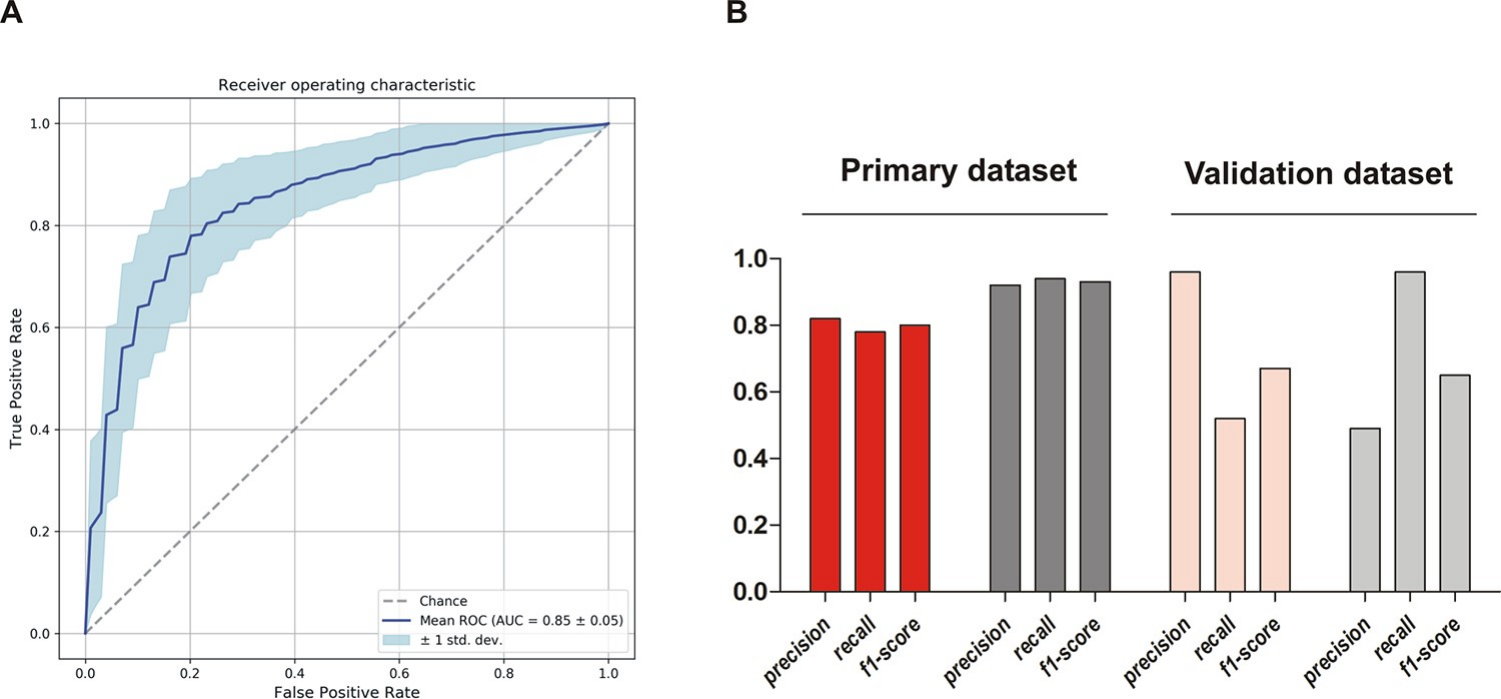
Performance parameters of k-mer-based prediction model. **(A)** Receiver operating characteristic curve (ROC-curve) of the final model including 96 k-mers associated with either infection or colonization. **(B)** Performance characteristics of the prediction model on the primary dataset and on the validation dataset from a previous study. Performance on infection isolates from this study (INF isolates) in red, performance on commensal isolates from this study (nCloNo isolates) in dark grey, performance on validation dataset in light red (infection isolates) and light grey (commensal isolates). Precision = (#True positive)/(#True positive + #False positive)(positive predictive value); recall = (#True positive)/(#True positive + #False negative)(sensitivity); f1-score = 2 * precision * recall / (precision + recall).

Employing the model on our isolate collection with which it was trained, it correctly assigned 18/23 invasive isolates and 58/62 commensal isolates (Recall 0.78 for INF isolates and 0.94 for nCloNo isolates, Fig 7). The precision of the model (positive predictive value) was 0.82 for INF isolates and 0.92 for nCloNo isolates. In order to test the model on an independent dataset, we selected 25 commensal strains from nasal swabs of healthy donors, isolated in Germany (isolated in 2003) and the UK (isolated in 2012), as well as 52 isolates from PJI (Germany, 2001–2003) from a previously published study[38]. In this validation dataset, 24/25 commensal strains were correctly assigned, while 27/52 infection-associated strains were correctly identified (Fig 7). Only one commensal strain was falsely assigned to the infection group but 25 infection isolates were incorrectly assigned to the commensal group. These results indicate that the model is highly specific but still has too restrictive criteria to robustly identify all infection isolates.

Overlap of the k-mer GWAS and the ORF-based GWAS was tested. 22/96 k-mers mapped with tolerance of one mismatch to the significantly associated genes from the gene-association study with a native p-value ≤0.05, only 14/96 k-mers mapped to genes from the gene-association study with a Benjamini-Hochberg adjusted p-value ≤0.05. This highlights the complementary nature of both approaches. Composition of the accessory genome, as analysed in the ORF-based GWAS is decisive for the armament of *S*. *epidermidis*, however, slight sequence variations as analysed by k-mer-based GWAS, both in core and accessory genome may influence gene function. Importantly, neither of the approaches can predict functional importance and associated sequences may also be co-inherited with other traits important for infection establishment.

### Intra-clonal heterogeneity and adaptation to the infectious lifestyle

The results from the comparison of INF and nCloNo isolates support the idea that certain phenotypes facilitate infection establishment. Therefore the hypothesis was put forward that beyond a general disposition of certain *S*. *epidermidis* strains, during transition from commensal to infectious lifestyle phenotypes conducive to survival in infection may further evolve and be selected for. To test this hypothesis, we identified nasal *S*. *epidermidis* isolates which were PFGE-identical to the INF isolate and thus likely represent descendants of the same ancestral strain from which the INF isolates emerged (referred to as clonal nose isolates [CloNo isolates]) [6,52,53]. CloNo isolates (median n = 3; minimum, n = 1, maximum n = 9) were found in 7/23 patients (patient HD04 [ST5], HD21 [ST2], HD26 [ST5], HD27 [ST5], HD29 [ST5], HD33 [ST87], HD59 [ST5], S2 Table). Previous studies have shown bacterial genetic diversification as a means of ensuring adaptability [54]. Therefore, in extension to analysis of adaptive events evident by comparison of CloNo and INF isolates, also bacterial diversification during infection was studied. To this end, multiple individual INF isolates per patient were analysed (median ten isolates per patient, minimum two, maximum ten) to represent *in vivo* population heterogeneity (Fig 1). Clonality of the selected isolates was confirmed in all infections by PFGE.

#### Phenotypic adaptation to infection promotes immune evasion and persistence

Phenotypes related to *S*. *epidermidis* pathogenesis were compared between CloNo isolates and INF isolates using a linear mixed model (LMM) with the individual patient as random factor. This statistical analysis takes into account the hierarchical structure of the data, i.e. isolate measurements are not independent, as within one patient isolates are more similar than between patients (due to deriving from the same genetic background, i.e. being PFGE identical).

**Fig 8:**
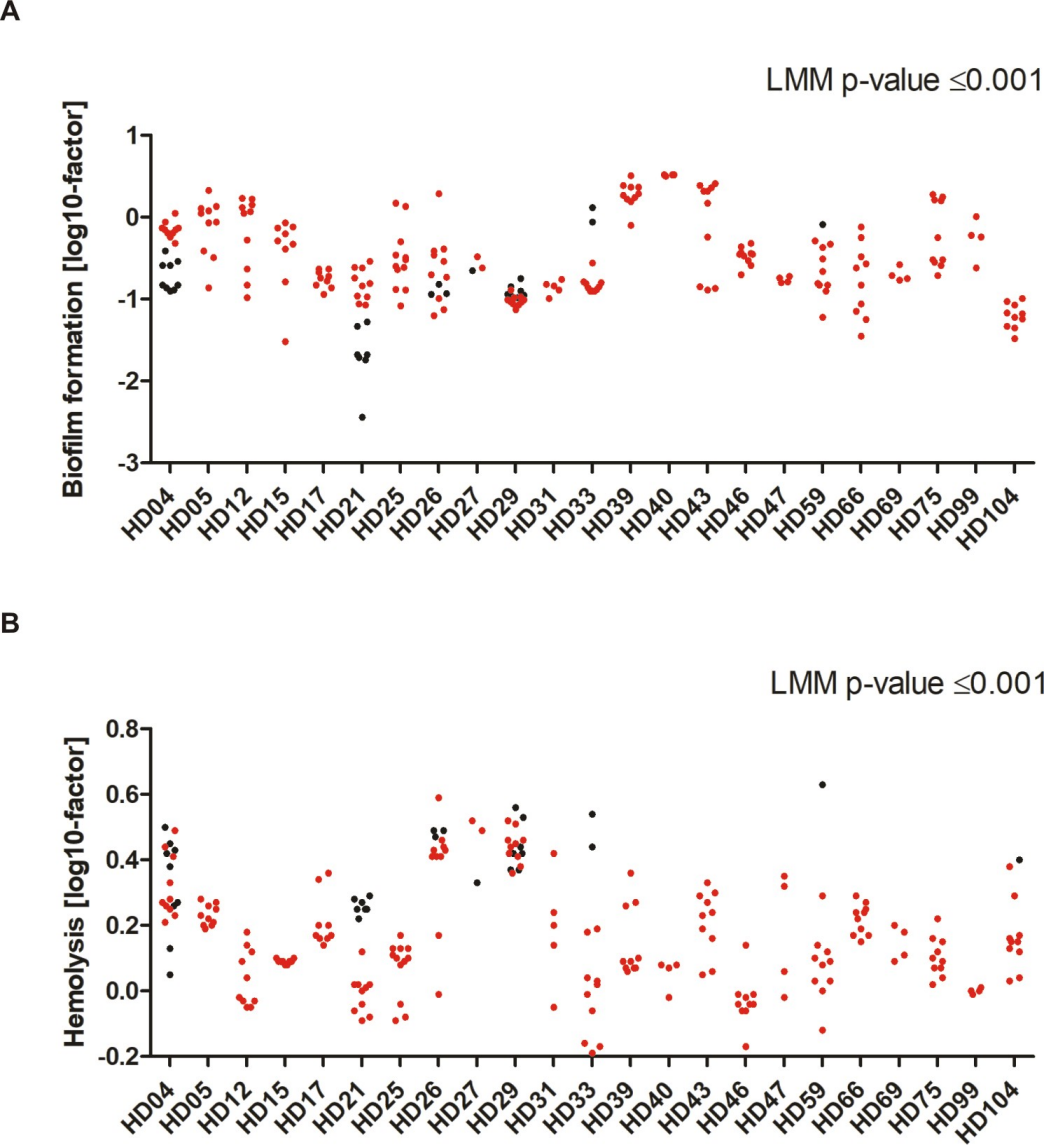
Distribution of biofilm phenotypes and hemolytic activity in invasive and commensal *S*. *epidermidis* populations metric variables (y-axis), sorted by patient (x-axis). Metric values were log transformed to attain symmetric distribution (indicated on y-axis label). Each dot represents one isolate. INF isolates are coloured in red, CloNo isolates are coloured in black. **(A)** Distribution of quantitative biofilm phenotypes produced in TSB. Plotted dots are means of biological duplicates. **(B)** Hemolysis of a 2% solution of sheep erythrocytes by supernatants of overnight cultures. Factor: extinction of free haemoglobin at 541nm in samples, normalized to hemolysis of only TSB. Plotted dots are means of biological triplicates.

Intriguingly, certain phenotypes were more pronounced in *S*. *epidermidis* INF than in CloNo isolates. INF isolates formed significantly more biofilm in TSB compared to CloNo isolates (p<0.001, factor 2.6, 95% CI: 1.69–4.08) (Fig 8A), and displayed less hemolytic activity (p<0.001, factor 0.6, 95% CI: 0.52–0.69) (Fig 8B). In addition, proteolysis of milk proteins was less commonly observed in INF isolates (p = 0.002, OR 0.064, 95% CI 0.012–0.35). INF isolates grew weaker in nutrient rich TSB (p = 0.001), however, when grown in nutrient poor RPMI and synthetic nose media, infection isolates showed stronger growth compared to clonally identical nose isolates (p = 0.001, factor 1.74; p = 0.01, respectively) (S6 Fig).

Growth in 70% pooled heat-inactivated human serum displayed no significant differences between CloNo and INF isolates (S6 Fig).

#### Intra-clonal diversity in antibiotic susceptibility is a common phenomenon in *S. epidermidis* PJI

MICs of oxacillin, vancomycin, rifampicin, penicillin, daptomycin and gentamicin were determined by gradient test, showing relevant MIC heterogeneity (difference > 2 dilutions) within INF isolates for gentamicin, penicillin and rifampicin, in some cases to an extent that would cause miscategorisation (S7 Fig), if only one single colony was subjected to susceptibility testing. For example, in 2/23 patients rifampicin MICs were elevated to “intermediate” according to EUCAST criteria in subpopulations. A subpopulation in patient HD26 carried a missense mutation in *rpoB* (D471G). In the other one no obviously relevant SNPs were detected.

**Fig 9:**
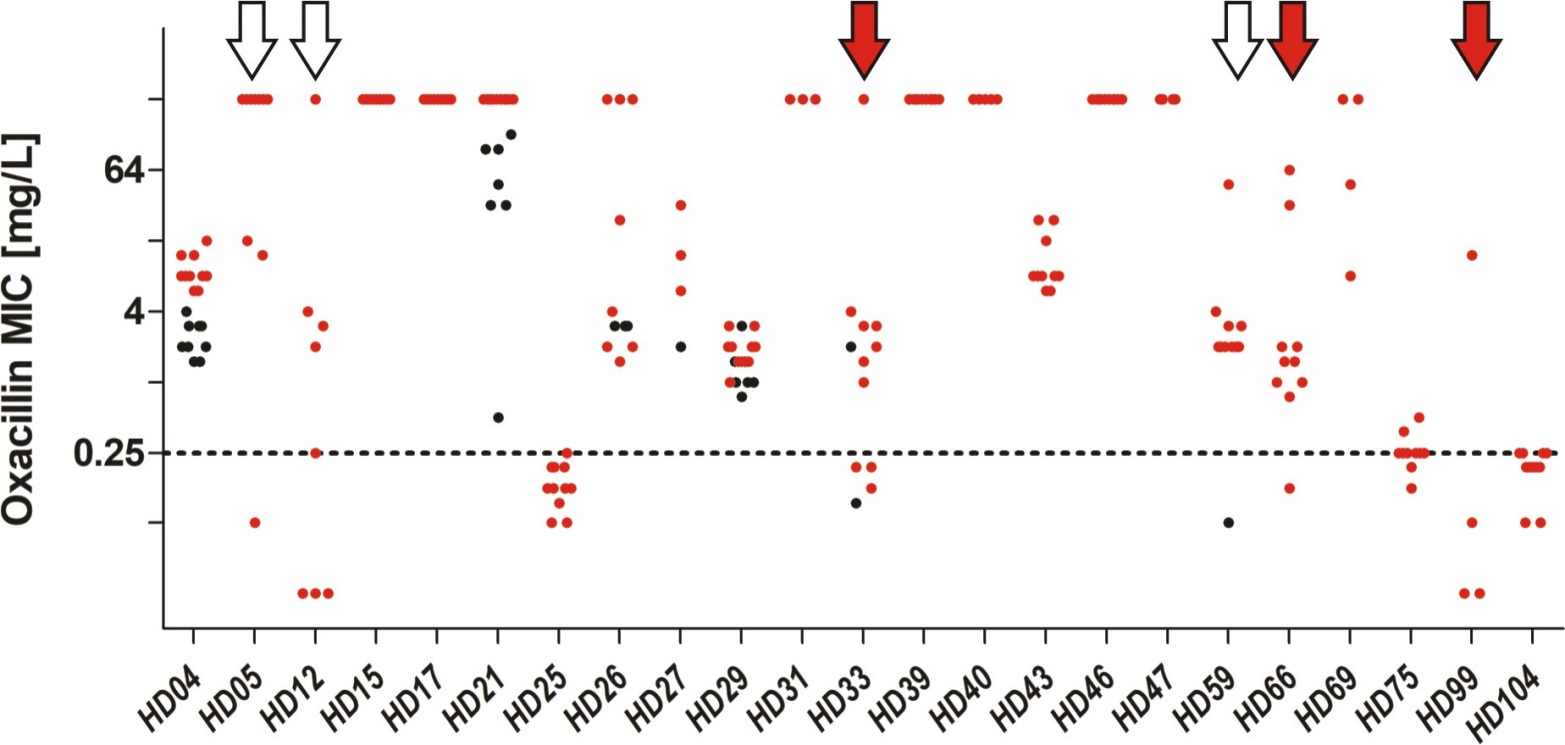
Distribution of Oxacillin MICs as determined by gradient test, sorted by patient (x-axis). INF isolates are coloured in red, CloNo isolates are coloured in black. Red arrows indicate cases with partial SCC*mec* deletions in a subpopulation. White arrows indicate cases where heterogeneity in PBP2A expression led to miscategorisation as susceptible in a subpopulation.

**Fig 10:**
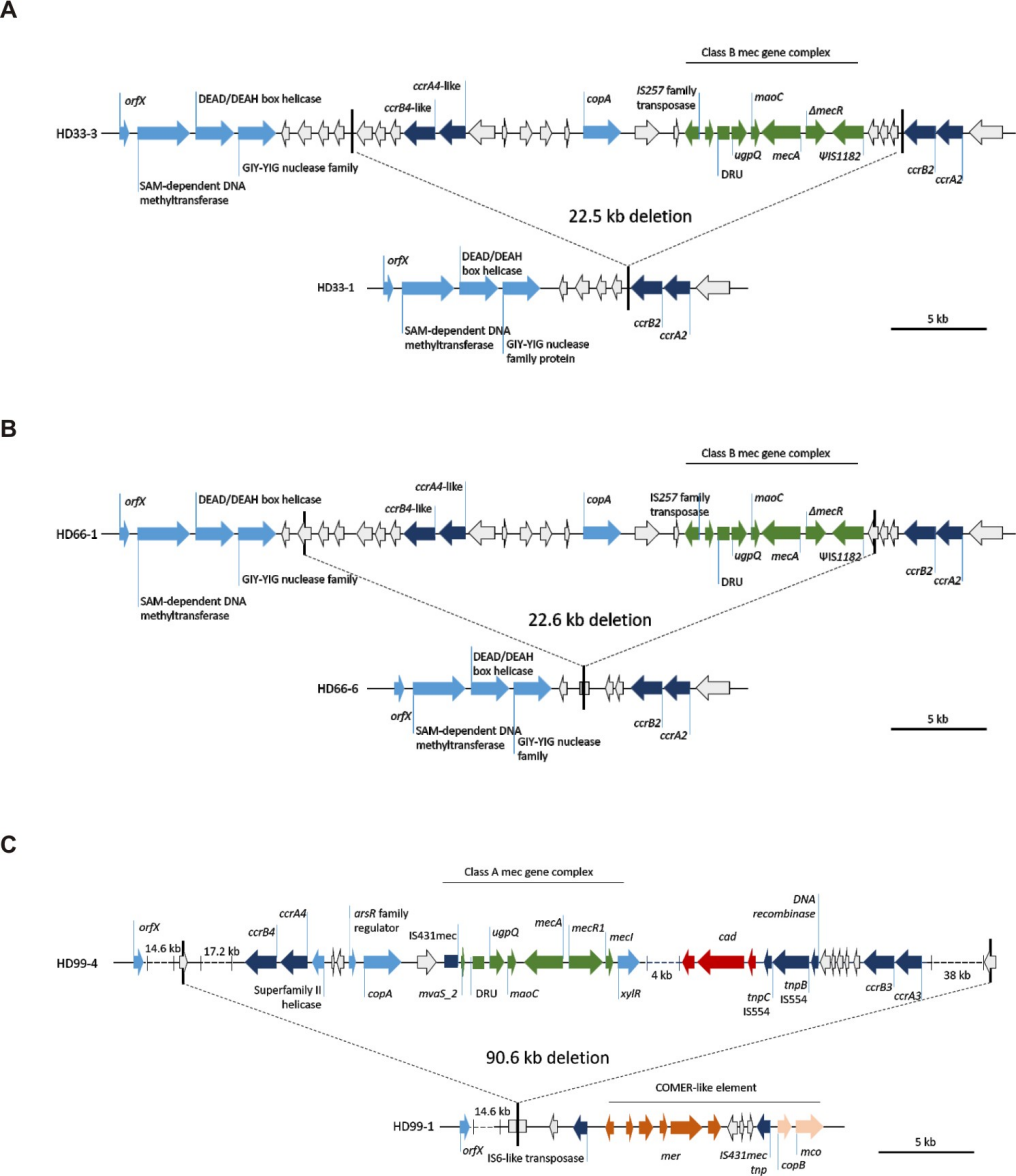
Gene map of deletion sites in the SCC*mec* element identified in *S*. *epidermidis* infection isolates. Elements were reconstructed based on hybrid assembly of MinIon and Illumina reads. **(A)** Patient HD33 **(B)** patient HD66, and **(C)** patient HD99. The 38kb fragment on the right contained a *ccrC* recombinase around 20kb downstream of the *ccrA3B3* operon. Each upper panel represents organization in respective isolates with complete SCC*mec*, lower panel shows organization with deletion. Green, *mec* gene complex; dark blue, recombinases; light blue annotated loci; white, hypothetical proteins; dark red, cadmium resistance operon; brown, mercury resistance operon; beige, copper resistance operon.

Most striking were the results of oxacillin susceptibility testing, identifying the parallel presence of both, susceptible and resistant isolates in 6/23 (21%) infections (Fig 9). In three of those, *mecA* was present in all isolates, but apparently expression was variable, with MICs ranging from below 0.25 mg/L (i.e susceptible) to ≥ 12 mg/L (i.e. resistant). More importantly, in three infections, deletions in the SCC*mec* element were identified in subpopulations (see section below and Fig 10).

#### Genetic diversification within infection populations

Phenotypic testing showed high intra-clonal variability in some patients. To further estimate the degree of within-host population heterogeneity, all INF isolates (2–10 per patient, 23 patients) and all CloNo isolates (1–9 per patient, 7 patients) were sequenced and analysed for genetic polymorphisms (total count INF isolates, n = 192; total count CloNo isolates, n = 30).

The analysis was performed to investigate the heterogeneity within the infection (INF group, n = 23) and to detect differences between INF isolates and their clonally identical CloNo isolates (INF-CloNo comparison, n = 7).

#### Major deletions occur in SCCmec and ACME during infection

**Fig 11:**
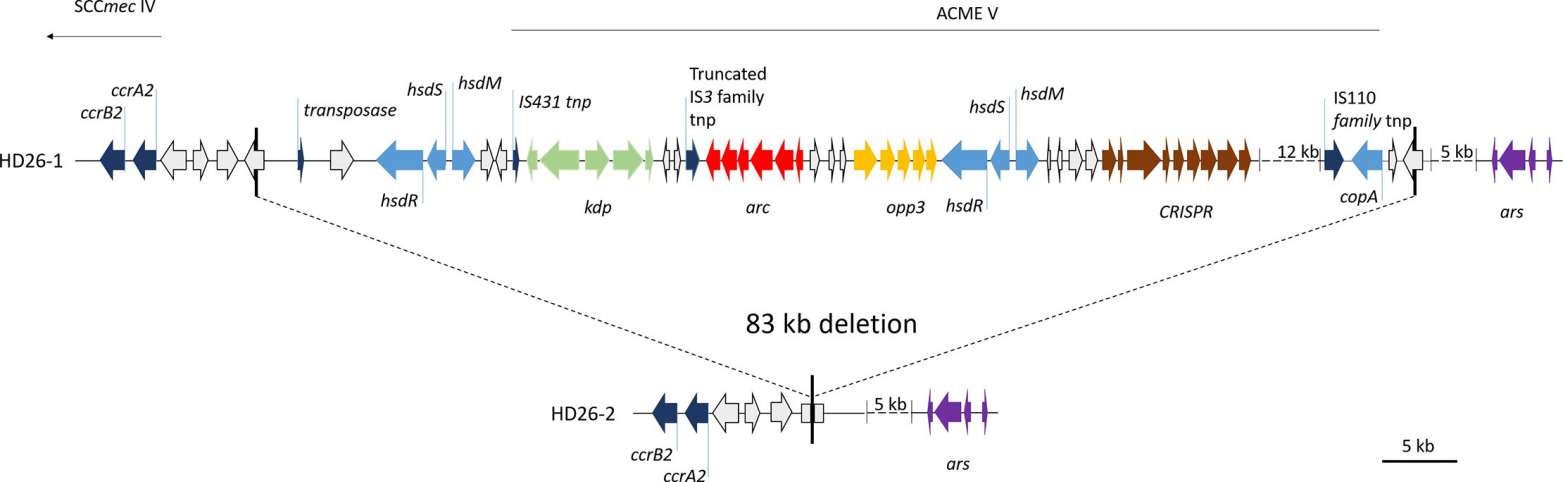
Gene map of deletion site in the ACME element in infection isolate from patient HD26. Elements were reconstructed based on hybrid assembly of MinIon and Illumina reads. Upper panel shows organization in *S*. *epidermidis* isolate with a complete ACME element, lower panel shows organisation after deletion event. Dark blue, recombinases; light blue annotated loci; white, hypothetical proteins; light green, *kdp* operon; red, arginine catabolism operon; yellow, oligopeptide permease ABC transporter *opp3* operon; brown, *CRISPR-Cas* locus; purple, arsenic resistance operon.

Major genomic rearrangements in the in SCC*mec* and ACME were observed within 4/23 INF groups, while these were found in 5/7 INF-CloNo comparisons. As reported above, deletions in the SCC*mec* element occurred in subpopulations of 3/23 INF groups (HD33 [ST87], HD66 [ST87], HD99 [ST2]) (Fig 10). Additionally, deletion of an arginine catabolic element V (ACME V) occurred in one infection (HD26 [ST5])(Fig 11).

In HD33 a 22,527 bp fragment containing partial type IV and composite *ccrA4* and *ccrB4* was deleted in 3/10 sampled INF isolates. *orfX* and *ccrA2* and c*crB2* remained in the chromosome, while the *mec* gene complex and *ccrAB4* were deleted. In HD66 a similar fragment of 22,637 bp was deleted in 1/10 sampled INF isolates. The deletion in HD66 was located 1396 bp further downstream compared to the deletion in HD33. The overlapping sequences of both deleted fragments were almost identical, except for four SNPs and a 102 bp insertion in HD66.

In HD99 (ST2) we found a deletion of a 90,568 bp fragment containing a composite SCC*mec* element. The content of *mec* gene complexes and multiple *ccr* gene complexes allowed no definitive SCC*mec* classification between composites of SCC*mec* type III(3A) or SCC*mec* type VIII(4A). In three of the four INF isolates, *orfX* remained as well, but the complete SCC*mec* element with adjacent regions was eliminated.

In patient HD26 a fragment of 82,976 bp, containing the ACME V element was deleted in one of ten analysed INF isolates.

The high plasticity of the *S*. *epidermidis* genome was highlighted by the comparison of PFGE-identical CloNo and INF isolates. Large genomic rearrangements were noted between INF isolates and CloNo isolates in patients HD21, HD29, HD33 and HD59. Interestingly, in HD21 a *ccrAB4*-containing fragment of a composite SCC*mec* element (type III(3A) and type VIII(4A)) was deleted from INF isolates. In HD33 (ST87) and HD59 (ST5), an ACME element containing a putative functional CRISPR-Cas system was deleted in INF isolates (ACME type V and I, respectively). While in HD29 only an ACME type V-associated fragment containing the *ars*-operon and a putative functional CRISPR-Cas system was deleted in INF isolates. This shows that rearrangements occur relatively often in the region of SCC*mec* and ACME compared to other regions (S10 Table), as has been observed *in vitro* [55–57]. Interestingly, deletions in these regions seem to occur preferentially in infection, while integrity is maintained in the nose. Loss of SCC*mec* in subpopulations within infection is of utmost relevance in a diagnostic setting: the release of susceptibility reports based on the testing of a single isolate can, in the worst case, lead to the false assumption of oxacillin susceptibility and thus lead to treatment failure.

#### The Agr system is a hotspot for within-infection variability

**Fig 12:**
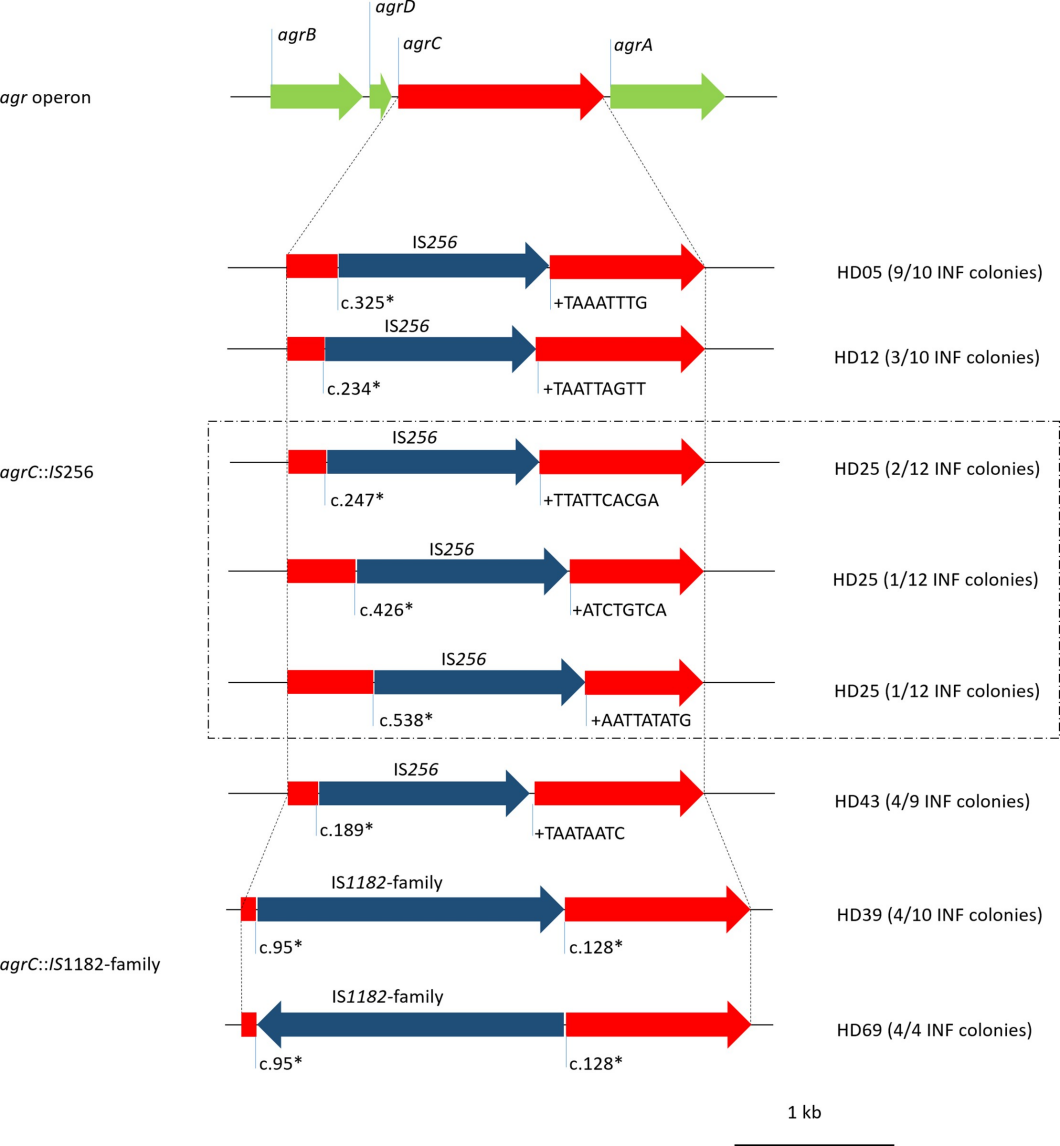
Gene map of IS insertions in *agrC*. Nucleotide numbering (*) according to reference sequence F1613_11135 from *S*. *epidermidis* ATCC 12228. Red, *agrC* and its fragments; dark blue, insertion sequences; light green, remaining *agr* operon. + sequences identify duplications at insertion site of IS*256*.

As mentioned above, loss of function in the Agr system has been associated with persistent infections, primarily in *S*. *aureus* [58]. Analysis of the *agrBDCA* locus provided evidence for insertion events in INF isolates from six patients. We identified IS*256* insertions in the receptor histidine kinase *agrC* at variable positions in subpopulations of four individual infections (HD05 [ST2], HD12 [ST2], HD25 [ST2], HD43 [ST23]). All insertions were flanked by 8-10bp direct repeats at the insertion sites. In one infection (HD25), insertion of IS*256* in *agrC* occurred at three independent time points, as we identified four subpopulations within the infection, one wild-type, one with an insertion at c.247 (reference sequence *agrC* from strain ATCC 12228, GenBank: QHG32737.1), one with an insertion at c.426 and one with an insertion at c.538. Moreover, IS*1182-*family insertions were noted in subpopulations of two infections (HD39 [ST297], HD69 [ST130]) (Fig 12). These insertions produced deletions of 34 bps at the insertion site. There was no significant difference in hemolysis, biofilm formation and growth rate between *agrC*-insertion and *agrC*-wt colonies overall analysed infections, as determined by linear mixed model. However, there was a tendency towards less hemolysis and more biofilm formation in two infections (HD05, HD43, S8 Fig).

#### Development of genetic within-infection heterogeneity is directed toward certain genes

Whole genome sequencing of multiple INF isolates revealed a mean of eight SNPs (range 0–45) in the coding regions per infection, cumulatively (S7 Table and S8 Table). The total number of non-synonymous and synonymous SNPs did not correlate with the patient-reported duration of symptoms or the time passed since implantation.

**Fig 13:**
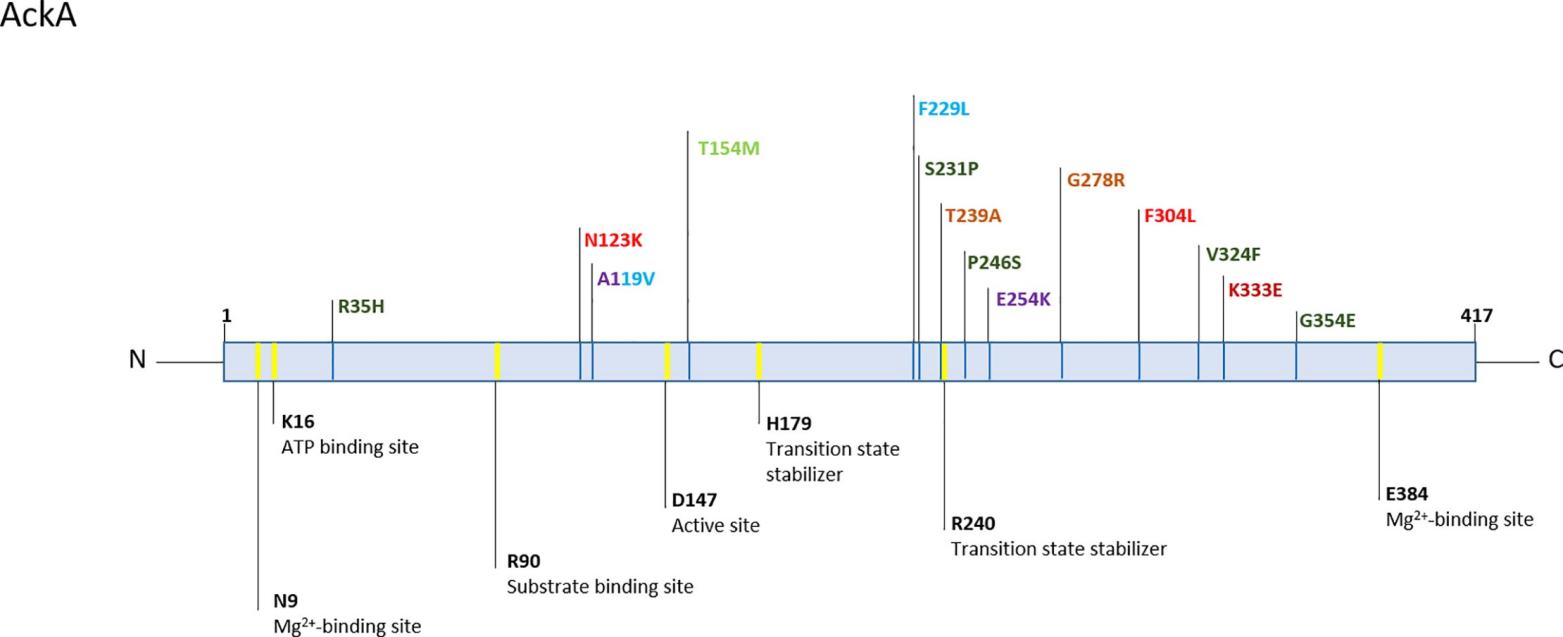
Schematic view of AckA protein. Sites important for enzymatic function as annotated by UniProt in yellow, annotation below. Variants are color-coded according to patient: HD15 purple [ST212], HD33 brown [ST87], HD39 light green [ST297], HD43 dark green [ST23], HD46 red [ST2], HD75 light blue [ST984] and HD99 dark red [ST2]. Reference AckA sequence from *S*. *epidermidis* ATCC 12228.

Intriguingly, some genes showed an enrichment of non-synonymous SNPs (nsSNPs). Most strikingly, the acetate kinase (*ackA*) was attainted by nsSNPs in 7/23 infections, five of which presented more than one polymorphism in the gene (Fig 13 and S11 Table). Certain mutations in *ackA* have been described to cause a defect in the phosphotransacetylase-acetate kinase pathway (Pta-AckA), a major pathway of energy generation in *Staphylococcus spp*. [59–61]. Interestingly, mutations were distributed over the entire protein, but seem to accumulate mostly 15 amino acids up- and downstream of D240, which is predicted as transition state stabilizer (Uniprot). We did not see any differences in growth in *ackA*-mutants compared to the isogenic clones from the same infection, neither under aerobic nor anaerobic conditions, neither in glucose-rich (TSB) nor glucose-free media (Bryant Burkey broth). However, the accumulation of mutations in *ackA* does not appear coincidental, and may play a role in directing energy acquisition from acetyl-CoA.

Besides *ackA*, we found an enrichment of mutations in the β-subunit of the RNA-polymerase (*rpoB*) with nsSNPs in 4/23 infections at positions F163I (HD05), S410F (HD43), D471G (HD26) and I956M (HD33). In HD26 the mutation D471G was associated with a relevant increase in rifampicin MIC in an INF isolate subpopulation. Mutation at the same amino acid position has previously been implicated in rifampicin resistance in *S*. *aureus*[62]. There was no effect on rifampicin MIC in the remaining infectious isolates.

#### Transcriptional changes during adaptation from commensalism to infection follow different trajectories

Though genome analysis provided clear genetic evidence for adaptive events during *S*. *epidermidis* progression to infection, these findings not fully explain the phenotypic shifts observed in invasive *S*. *epidermidis* isolates in comparison to corresponding nose commensal isolates. In order to further investigate adaptive processes from commensalism to infection, gene expression profiles of INF isolates and their corresponding CloNo isolates (INF-CloNo comparisons) were analysed in five individual patients (HD04, HD21, HD26, HD29, HD33). Patients were selected based on whether at least two CloNo isolates were available to allow for meaningful statistical analysis. Two INF isolates and two corresponding CloNo isolates per patient were selected. Triplicates of each isolate were grown in TSB and 50% heat-inactivated human serum in phosphate-buffered saline (50% hS) for 6h to exponential phase and RNA-Seq experiments were performed.

*S*. *epidermidis* strain HD04 (chromosome: CP052985, plasmid 1: CP052986, plasmid 2: CP052987 [ST5]) was selected as reference and p-values were adjusted for multiple testing using the Benjamini-Hochberg procedure[63].

**Fig 14:**
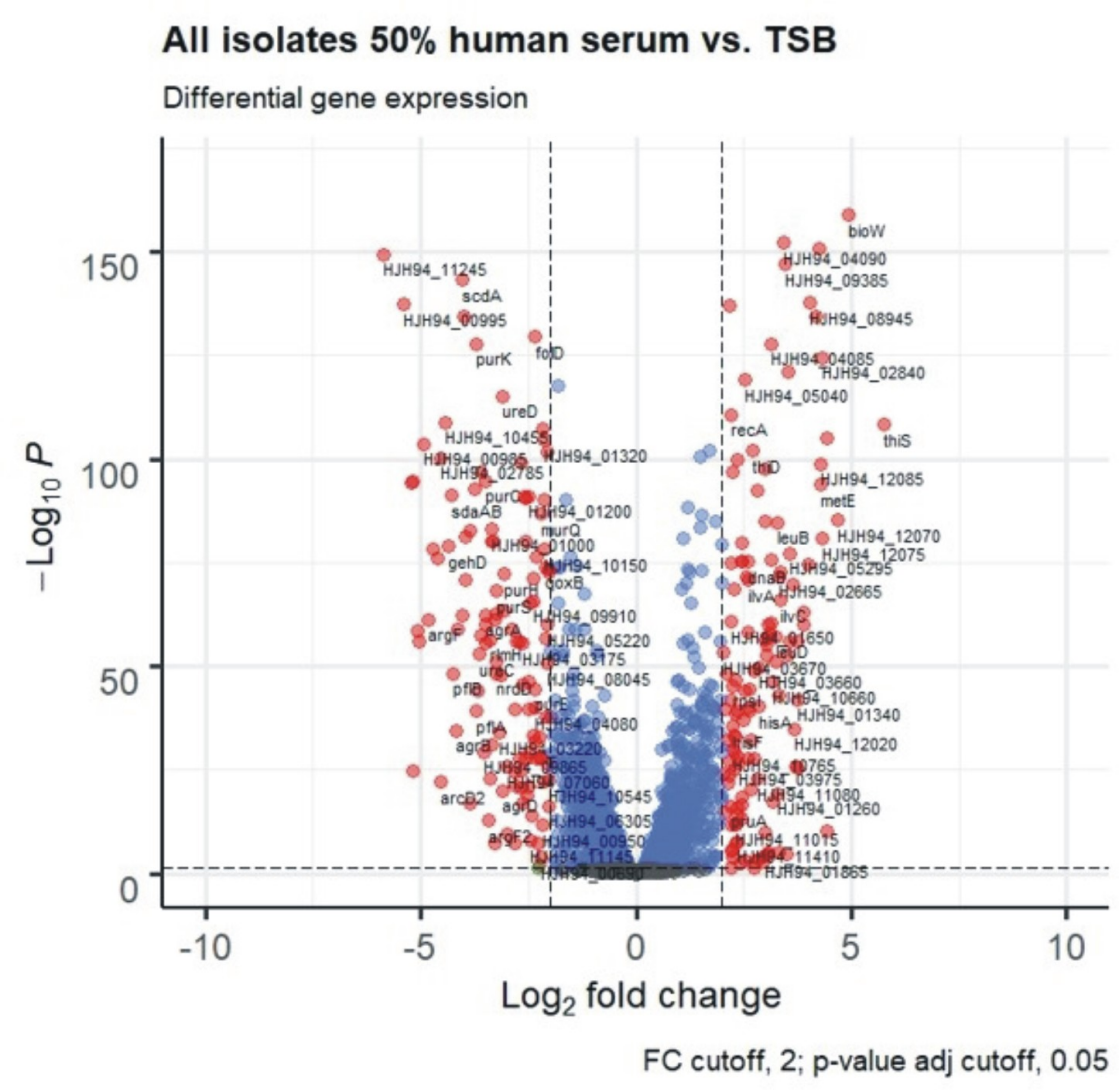
Volcano plot of RNA-Seq of all isolates in 50% human serum versus TSB after 6h of growth. Red dots signify genes with log_2_-fold-changes ≥2, and a Benjamini-Hochberg procedure adjusted p-value ≥0.05. Blue dots are genes with adjusted p-value ≥0.05, green dots are genes with log_2_-fold-changes ≥2. Genes represented by grey dots fulfil neither condition.

Importantly, gene expression during growth in 50% hS compared to the standard media TSB was vastly different. Pooled expression data from all isolates showed that 74% of all genes (1716/2322 genes, adjusted p-value ≤ 0.05) were differentially expressed, illustrating the immense capacity of *S*. *epidermidis* to adapt to different conditions (Fig 14). There was no preference for functional groups (Cluster of Orthologous groups, COG categories) among differentially expressed genes. Interestingly, the Agr-quorum sensing system (*agrBDCA*, fold change -9.5 to -17, adj. p<0.001) and genes encoding PSMs (*psm*β1a, *psm*β1b, *psm*β2, *psm*β3 Fold change -5.6 to -10.2; *psm*α, fold change -16, *psm*δ, fold change -9; adj. p<0.001), their exporting system (*pmtABCD*, fold change -10 to -11, adj. p<0.001) and the delta-hemolysin, encoded in the Agr-effector RNAIII, (fold change -16, adj. p<0.001) was strongly down regulated during growth in 50% hS. These genes belonged to the set of genes exhibiting the most variable expression between growth in 50% hS and TSB, similar to findings in the related species of *S*. *aureus*[64].

Comparing gene expression between TSB-grown INF and CloNo isolates from the same patient, differentially expressed genes (DEGs) were found in 3.8%, 34.2%, 34.5%, 25.2% and 7.8% of total genes from patients HD04, HD21, HD26, HD29 and HD33, respectively (S9 Fig). The corresponding values were 3.6%, 34.2%, 21.6%, 19.4% and 4.8%, for strains grown in 50% hS. No gene was found to be differentially expressed in all INF-CloNo comparisons and the number of genes differentially expressed in more than one INF-CloNo comparison was very low. Thus, no obvious uniform adaptation program can be deduced from gene expression patterns. The great variability of gene expression in the INF-CloNo comparisons was surprising considering that INF and CloNo isolates are only separated by a relatively small number of SNPs (mean: 44 nsSNPs, range: 0–110, S9 Table). However, the genetic rearrangements of variable size observed in HD04, HD21, HD29 and HD33 may affect the transcription of the whole organism (S10 Table).

#### *Regulation of gene expression of the* Agr*-system may contribute to adaptation in the absence of specific mutations*

Previous studies in both *S*. *epidermidis* and *S*. *aureus* found mutations in *agr* to mediate adaptation to the infection environment and reduced virulence in prolonged infections [43,65]. Though we did not find any of mutations in *agr*-genes, we noted differential expression of *agr* and *psm*s between INF and CloNo isolates in HD21, HD26, HD29 and HD33 (S12 Table). Interestingly, while RNA-Seq results from HD26 and HD33 support a direct regulation of *psm*-genes by *agr* [66], in HD21 *agr*-genes are up-regulated while *psm*-genes were downregulated, indicating hitherto unrecognized regulatory mechanisms in *psm* transcription. Along this line, in HD29 no differences in *agr* expression is noted between INF and CloNo isolates, while *psmβ* genes are up-regulated in INF isolates.

## Discussion

*S*. *epidermidis* is an important pathogen in foreign-material associated infections in vulnerable hosts. In order for this harmless commensal species to successfully transition from its natural reservoir to the hostile environment in infection, bacterial adaptation processes must occur. We established a patient-matched collection of commensal and PJI-associated *S*. *epidermidis* isolates and present data demonstrating that important adaptive events occur on both phenotypic and genotypic levels during establishment of chronic *S*. *epidermidis* PJI.

The rigorous inclusion of only simultaneously collected commensal and invasive *S*. *epidermidis* from well-characterized PJI patients allowed for a robust evidence base in the comparison of infection-associated strains and clonally diverse commensal strains. In addition to previously reported key phenotypes associated with *S*. *epidermidis* invasiveness, most importantly biofilm formation [13,15,16], novel insights into overarching phenotypes facilitating survival during infection (e.g. reduced cytolysis, enhanced growth in nutrient-poor media) were obtained. The growth advantage of *S*. *epidermidis* infection isolates in iron-free and nutrient-poor media indicates that these are better equipped for sustained growth in infection as nutrients typically get depleted by acute phase proteins in the host’s attempt to starve the pathogen[67,68]. Of notice, these phenotypes were at least partially linked to ST2 and ST5, which were overrepresented in infection.

In recent years, novel hypothesis-free computational approaches have been employed in the study of genetic factors associated with *S*. *epidermidis* invasiveness or with infection outcome [38,69]. We here employed two complementary computational approaches based on ORFs and k-mers to identify predictors for infection in this small but highly defined isolate collection. Previous genomic studies of *S*. *epidermidis* often include a great variety of infection types and mostly commensal control groups from healthy donors. This may lead to a certain bias as community-associated *S*. *epidermidis* populations differ from those in the hospital where most infections are acquired [18,30].

Indeed, findings from both computational approaches reinforce the importance of mobile genetic elements in the pathogenic potential of *S*. *epidermidis* [39,70,71]. A striking 94% of genes associated to infection in the ORF-based GWAS unambiguously matched to the mobilome. Besides previously described mobile genetic elements, such as SCC*mec* and IS*256*, we identified three infection-associated prophage regions which were predominantly found in ST2. Importantly, these were also present in ST2 *S*. *epidermidis* from an international collection of *S*. *epidermidis*, substantiating the potential biological importance during infection [15]. Indeed, phages are crucial drivers for bacterial evolution and pathogenicity. Defined prophages from *Escherichia coli* K12 increase bacterial fitness, and temperate prophages in *Pseudomonas aeruginosa* and *E*. *coli* were found to support biofilm formation by promoting eDNA release through activation of lytic genes under stress conditions[72,73]. In *Neisseria meningitidis* the production of extracellular phage-filaments promotes host cell colonization [74]. In *Staphylococcus aureus*, a closely related species to *S*. *epidermidis*, prophages increase virulence of their bacterial host, most probably by promoting interaction with extracellular matrix components or by integration of phage-encoded virulence determinants into the staphylococcal genome [75,76]. Future studies will therefore need to address whether prophages may at least partly explain the over-representation of ST2 in *S*. *epidermidis* infections, and the recently reported international spread of the ST2.

It is evident that some clonal lineages and the presence of a variety of genetic determinants can influence *S*. *epidermidis* pathogenicity, still 20–60% of all *S*. *epidermidis* infections are caused by clonally diverse strains that are equipped with only a subset of established or even no known pathogenicity factors[77]. It can therefore be speculated that most *S*. *epidermidis* strains possess the inherent ability to establish infection in a vulnerable host and that the remarkable adaptive potential of *S*. *epidermidis* is at the basis of its survival during invasion and establishment of chronic infection.

In this cohort of PJI patients, we were able identify commensal isolates PFGE-identical to the infection strain in 7/23 studied cases, allowing to gain insights into intra-clonal changes associated with adaptation to infection. Phenotypes changed at the transition to infection in a direction fit to improve immune evasion (i.e. increase in biofilm formation, reduced cytolytic activity, enhanced growth in iron-free and nutrient poor media). Furthermore, we found evidence for streamlining of genetic content in infection by deletion of SCC*mec* and ACME fragments in 5/7 pairs of infection isolate and the clonally-identical nasal isolate. The hypothesis that some gene content from the hyper-variable region of ACME and SCC*mec* may be a burden in infection and only confer an advantage in some conditions on the human skin is further substantiated by the finding that in 4/23 studied infection populations genetic heterogeneity with partial deletions in SCC*mec* and ACME emerged. The driving force behind these rearrangements remains unclear. Anecdotal reports found partial SCC*mec* loss in *S*. *aureus* possibly during infection or after [78,79], and *in vitro* passaging experiments suggest that SCC*mec* deletions can be induced by exposure to vancomycin [80,81]. Also in *S*. *epidermidis*, two case reports documented deletions within SCC*mec* during infection [82,83].

Given that standard methods in resistance testing rely on examination of single colonies, the regular emergence of intra-clonal SCC*mec* heterogeneity in *S*. *epidermidis* could lead to significant diagnostic errors, where incorrect assumption of oxacillin susceptibility may lead to unfavourable treatment outcomes.

Previously, the importance of insertion sequences in staphylococcal genetic variability has been reported, for example IS*256* insertion in *icaADBC*, *sarA* or *rsbU* leading to phase variation of biofilm formation *in vitro* [84,85]. Importantly, we identified insertion sequences in the sensor histidine kinase of the quorum sensing system (*agrC*) in a total of six infections. Interestingly, in all but one only a subpopulation was attainted with loss of function of the Agr system.

Independent studies in *S*. *epidermidis* and *S*. *aureus* have identified loss of function mutations within the master virulence regulatory system Agr, particularly located in *agrA* and *agrC*, during blood stream infections and endocarditis [43,86]. In *S*. *aureus* these mutations appear to promote a chronic course of infection, enhancing biofilm formation and possibly furthering immune evasion strategies [87]. However, Agr-defects come at a cost in *S*. *aureus*, for example, virulence of Agr-deficient strains was reduced in a skin infection animal model and the pathogen’s ability to lyse neutrophils is impaired [87,88]. The Agr-system in *S*. *aureus* and *S*. *epidermidis* share similarities, but are by no means identical. Data on the function of the Agr-system during infections of *S*. *epidermidis* is still scarce. We did not find a clear phenotype associated with the off-variant, albeit two of six infections displaying both variants showed a dichotomy of hemolysis and biofilm phenotypes, as one might expect.

Strikingly, when we analysed gene expression to compare infection isolates and PFGE-identical nasal isolates, the quorum sensing Agr-system or its most prominent effectors were also differentially expressed in four of five comparative analyses. Both up- and down-regulation of *agr*-genes in infection isolated was observed, while also a strikingly variable response of the directly regulated transcription of genes encoding PSMs was noted. These findings support the idea that in *S*. *epidermidis* alternative regulatory networks of cytolytic and biofilm-structuring PSM peptides exist [89].

Based on the formation of subpopulations with phase variation in *agrC* and the changes in gene expression during infection, it appears reasonable to assume that both an increase and decrease of Agr-activity goes along with certain gains and trade-offs for the pathogen in infection. It will be of great interest to identify the drivers selecting for either state in the future.

Besides larger genomic rearrangements resulting in a reduction of gene content, the repeated formation of subpopulations with variants in *ackA*, coding for actetate kinase and *rpoB*, coding for the β-subunit of the RNA-polymerase (7/23 and 4/23 infections, respectively) indicates that genetic heterogeneity and within-host evolution at the gene level is important during chronic *S*. *epidermidis* infection. Previous reports suggested genetic heterogeneity as a bet-hedging strategy, preparing bacterial populations for future changes in conditions [90,91]. However, we believe that the importance of micro-niches within infection may be underappreciated. Nutrient supply and exposure to the immune system vary depending on whether bacteria grow planctonically in synovial fluid or embedded in a biofilm architecture. Moreover, evidence indicates that within biofilms adherent to the implant and the adjacent bone nutritional and oxygen gradients develop [92]. These differing conditions may be drivers behind genetic diversification within infections, and evolution of both, *ackA* and *rpoB*, could support *S*. *epidermidis* micro-niche adaptation. For example, certain mutations in *ackA* have been described to cause a defect in the enzyme’s activity of converting acetyl-CoA to acetate, leading to diversion of carbon flux to alternative metabolic pathways, i.e. the TCA cycle or glycolysis [59–61]. Mutations in *rpoB* are known to change expression profiles in some bacterial species such as *Mycobacterium tuberculosis* and *E*. *coli*, thus polymorphisms in the gene may contribute to a plethora of diverse phenotypes [93,94]. It will be of great importance to study the impact of the identified polymorphisms on *S*. *epidermidis’* ability to invade into certain micro-milieus. Models more closely resembling the infection site need to be developed in order to fully appreciate the challenges *S*. *epidermidis* faces during infection.

### Limitations of the study

We did not sample different body sites to collect commensal *S*. *epidermidis* isolates. We chose to sample the patients’ noses, due to its high bacterial density and protection from skin disinfectants during the course of the hospital stay. Obviously, *S*. *epidermidis* occurs at several different cutaneous body sites, sampling of which may have helped recover more matched pairs of commensal and infection associated isolates. However, transfer of *S*. *epidermidis* strains from one cutaneous niche to another occurs frequently, and anatomical regions, except for the navel and foot, appear to be of similar population composition [95].

We relied on PFGE to determine clonal relatedness of invasive and commensal isolates. As of now, there is no absolutely incontestable way to prove that these infection and commensal isolates emerged from the same progenitor in the respective patient and that differences between the nasal and infection clones indeed demonstrate adaptation to the respective niche. PFGE has been the gold standard to determine clonal relatedness in staphylococci for many years and we here rely on this tried and tested method [35,52]. The advent of WGS brought more discriminatory power to analyses, however, we still lack clear criteria to determine clonal relatedness in *S*. *epidermidis*. The polyclonal nature of *S*. *epidermidis* populations on the skin as well as the species’ proneness to major genetic rearrangements promotes acquisition and loss of genetic material [95]. Important changes in gene content may occur in populations separated only a short while ago. However, WGS schemes for determination of clonal relatedness (e.g. cgMLST) mostly rely on SNP calling in the core genome and thus may underestimate differences in gene content that may be uncovered by PFGE.

We identified commensal isolates, clonally identical to the invasive one, in seven out of 23 infections. This proportion might have been increased with a prospective study starting at the time of first implantation of the prosthesis, as most PJI occur through inoculation around this time. However, as infection rates are comparably low at <2%, over 5000 patients would have to be sampled and followed-up in order to recruit an equal number of *S*. *epidermidis* infections as we did in this study.

The matched commensal/invasive pairings which we found mostly belong to ST5, while overall ST2 was the most important ST in infection. Moreover, isolates belonging to ST2 were rarely found in the group of commensal nCloNo *S*. *epidermidis*, putting forward the question from which reservoir the infection-causing ST2-related strains derive. On one hand, it is conceivable that ST2 is indeed a hospital adapted *S*. *epidermidis* line, circulating within health-care facilities, being transferred from fomites or health care personnel to a patient to subsequently cause foreign material-associated infection [96,97]. Our recovery of commensal ST2 may also have been limited by only sampling the patients’ nares. Future studies will need to investigate if niche specific colonization pattern and over-representation of defined clonal lineages at anatomical sites (e.g. dry, sebaceous areas) are related to the clonal organization of invasive *S*. *epidermidis*.

## Conclusions

In conclusion, our data lend support to the idea of a virulence continuum in *S*. *epidermidis* strains characterized by the combination of different phenotypic and genotypic traits, which are partially, but not exclusively, associated with defined clonal backgrounds. Apparently, during pathogenesis of PJI *S*. *epidermidis* can make use of its exceptional ability to flexibly adapt to novel niches. Phenotypic and genotypic diversification during infection is a common event with immediate implications for microbiological diagnosis and treatment of *S*. *epidermidis* PJI.

## Material and methods

### Ethics statement

The study was approved by the Hamburg Ethic Commission (number PV4892). Formal written consent was obtained from all participants.

### Biofilm formation

Strains were inoculated into 2mL of tryptic soy broth (TSB) from blood agar plates and incubated at 37°C for 6h. Cultures were then diluted 1:100 in TSB and TSB supplemented with 4% NaCl. 200μl of the suspension were then transferred to a 96-well cell culture plate (Nunclon Delta Surface, Thermo Fisher Scientific, Waltham, MD, USA) and incubated for 18-20h at 37°C under static growth conditions. OD_600_ was recorded and supernatant media was then removed and the remaining biomass gently washed three times with sterile PBS in order to remove non-adherent cells. 96-well plates were left to dry and then stained with 100μl of crystal violet per well for 10min. Wells were washed again and Biofilm formation was quantified by assessing the absorbance at 570 nm and 405 nm as a reference wavelength. Experiment were performed in duplicates with four technical replicates each.

The stability of the biofilm phenotype was tested on a subset of two isolates each from six infections and was maintained over 12 generations. Thus, the phenotype of each analysed clone is stable and heterogeneity is not due to stochastic fluctuations.

### Hemolysis

Strains were inoculated into 2mL of TSB from blood agar plates and incubated at 37°C for 6h. Cultures were then diluted 1:1000 in 10mL of TSB and incubated for 16-18h at 37°C, 200rpm. Cells were then pelleted by centrifugation at 3500g for 10 min. 1.5mL of supernatant was aliquoted and stored at -20°C until further use. A sheep erythrocyte solution was prepared by diluting a 50% blood suspension in Alsever-buffer (Labor Merck, Würzburg, Germany) 1:25 in PBS to a final concentration of 2%. 100μl of the erythrocyte solution and 100μl of culture supernatant were then mixed and incubated at 37°C for one hour under gentle agitation. As internal reference the erythrocyte solution was mixed with pure TSB and as positive control the solution was mixed with TSB+1%Tween. After one hour of incubation, samples were centrifuged at 13 000rpm in a table centrifuge and 150μl of supernatant were transferred to a 96-well flat-bottom plate (Greiner Bio-One, Kremsmünster, Austria). Absorption at 541nm as a measure of free haemoglobin in the supernatant was read on an ELISA reader (Tecan infinite M200, Männedorf, Switzerland). Absorption was then normalized to absorption of the internal reference. Experiments were conducted in triplicates. Biological replicates were averaged for statistical analysis.

### Growth analysis

Strains were inoculated into 2mL of TSB from blood agar plates and incubated at 37°C for 6h. Cultures were then diluted 1:1000 in either 2mL of TSB (BD, Heidelberg, Germany), TSB +4% NaCl, RPMI 1640 (Gibco, Thermo Fisher Scientific), 70% heat-inactivated human serum (pooled from 18 healthy individuals, diluted with 30% phosphate-buffered saline), permission for the use of anonymous donations (WF-015/12) was obtained from the Ethical Committee of the Ärztekammer Hamburg (Germany), and synthetic nasal media (SNM3)[34], 200μl of the suspension were inoculated into 96-well flat-bottom plates (Greiner, Bio-One, Kremsmünster, Austria) and incubated for 22h at 37°C in an ELISA reader (Tecan infinite M200, Männedorf, Switzerland). OD_600_ was automatically determined after brief agitation every 30min. Experiments were conducted in duplicates. Biological replicates were averaged for statistical analysis. Growth curve AUCs were determined in GraphPad Prism v5 (GraphPad Software, La Jolla, CA, USA).

### Susceptibility testing

Susceptibility testing was conducted according to the manufacturer’s instructions on a Vitek 2 instrument with the GP67 AST card (Biomérieux, Marcy l’Etoile, France). Minimal inhibitory concentrations (MIC) were determined by gradient diffusion strip on Müller-Hinton agar (Oxoid, Thermo Fisher Scientific, Waltham, MD, USA) for oxacillin, vancomycin, rifampicin, gentamicin, daptomycin and penicillin (Lioflichem, Roseto degli Abruzzi,Italy; Biomerieux, Marcy l’Etoile, France). Etests were incubated at 37°C in ambient air and read after 20h. Interpretation of results was according to EUCAST criteria.

### Proteolysis

Skim milk agar was prepared as described previously [98]. Strains were inoculated into 2mL of TSB from blood agar plates and incubated to an OD_600_ of 0.4. 10μL of the bacterial culture were inoculated on a 12mm filter disk placed on the skim milk agar. Proteolysis zones on the agar were measured after 24h and 48h. For analysis proteolysis was determined as either positive (proteolysis zone around filter disk clearly visible) or negative, as measured diameters did not follow a symmetric distribution. Experiments were conducted in duplicates. Biological replicates were averaged for statistical analysis.

### Pulsed-field gel electrophoresis

All isolates were subjected to PFGE, in order to test clonality of the isolates from infection (INF isolates) and to type corresponding nasal isolates of the same patient in relation to the infectious isolate. Nasal isolates which showed patterns identical to the infectious isolate were assigned as CloNo isolate (clonal nose isolate). Isolates showing patterns that differed in PFGE by at least one band from the infectious isolate were assigned as nCloNo isolate (non-clonal nose isolate). Of the nCloNo isolates, only one isolate per PFGE-pattern was selected for further analysis.

PFGE was performed by a modification of the method described previously [99], using a contour-clamped homogenous electric field (CHEF DR III, BioRad, Hercules, CA, USA). Bacterial cells were grown overnight in TSB, pelleted and poured into low melting point agarose blocks. The cell wall was digested with recombinant lysostaphin (Sigma Aldrich, St. Louis, USA). Restrictions was performed with the endonuclease SmaI (Thermo Fisher Scientific, Waltham, MD, USA). Pulse time was 5.0–50.0 s for 20h. DNA restriction patterns obtained by PFGE were interpreted manually according to the criteria published previously[100]. One band difference was accepted for clonally related isolates.

### Statistical analysis of phenotype

#### Comparison of INF and nCloNo S. epidermidis isolates

In order to compare the groups of infection-associated (INF, n = 23) and commensal isolates (nCloNo, n = 62), a virtual INF isolate for each infection was calculated by averaging the values of phenotype variables of all INF isolates from one infection (2–10 INF isolate per patient -> compiled into one virtual isolate by averaging values of variables -> one virtual isolate per infection goes into comparison with nCloNo isolates, as not to introduce a bias into the comparison by adding more than one isolate per infection). Phenotypes were tested for a symmetric distribution and transformed to a logarithmic scale in order to attain normal distribution where appropriate. Metric variables were analysed by Student’s *t-*Test. Categorical variables were tested by Pearson’s chi-square. Statistical significance was accepted at a *p-*value ≤0.05. Analyses were conducted in SPSS version 25 (IBM, Armonk, NY, USA). Plots were created in R with the ggplot2 package[101] and GraphPad Prism v5 (GraphPad Software, La Jolla, CA, USA).

#### Analysis of intra-clonal phenotypic adaptation

In order to compare phenotypes of INF isolates CloNo isolates, we used mixed models with the individual patient as random intercept. To analyse metric variables, a linear mixed model was used. Variables were log_10_-transformed where appropriate, in order to attain symmetrically distributed values and residues. Results are reported as either mean effect size between INF and CloNo isolates or in case of log-transformed variables, as a factor (10 to the power of co-efficient). *P*-value and 95% confidence interval of mean effect size or factor are given. Categorical variables were analysed in a mixed ordinal regression model. Results are reported as odds ratio (OR), 95% confidence interval of OR and p-value. Statistical significance was accepted at a *p-*value <0.05. Analyses were conducted in SPSS version 25 (IBM, Armonk, NY, USA).

### Whole genome sequencing

Illumina short read sequencing was performed for all INF isolates (2–10 per infection, 23 infections, n = 194), CloNo isolates (n = 30) and nCloNo isolates (n = 62).

Strains were inoculated into 3mL of TSB from blood agar plates and incubated at 37°C under vigorous shaking for 4-6h. Cells were pelleted, washed with phosphate-buffered saline and mechanically lysed with zirconia beads 3x20s on a tissue homogenizer (Precellys 12, Bertin, Montigny-le-Bretonneux, France). DNA was then extracted with the QIAamp Mini Kit (Qiagen, Hilden, Germany) according to the manufacturer’s instructions. DNA was then fragmented on a Bioruptor Pico instrument (Diagenode, Seraing, Belgium) to a fragment length of approximately 300-400nt. DNA libraries were constructed with the NEB Next Ultra DNA library Prep Kit for Illumina (New England Biolabs, Ipswich, MA, USA) and whole genome sequencing was conducted on a NextSeq 500 sequencing system and a 300-cycle mid-output kit (Illumina, San Diego, CA, USA). A mean of 4.3 million paired-end, 150 nt reads were generated. Bases less than Q30, as well as adapter sequences of the reads, were trimmed and any reads shorter than 35 nt were removed using Trimmomatic v0.36 [102].Retained high-quality NextSeq reads were used as input for the SPAdes assembler (version 3.7.1) [103], resulting in a mean depth of 250.3 (max: 1487.9, min: 10.6) and an N50 contig length of 123 kb.

Long-read Nanopore sequencing (Oxford Nanopore technologies, Oxford, UK) was performed for at least one isolate per infection and all isolates with SCC*mec* or ACME rearrangements (HD04-1, HD05-1, HD12-1, HD17-1, HD21-2, HD21N4, HD25-1, HD26-1, HD26-2, HD27-2, HD27N1, HD29-1, HD31-1, HD33-1, HD33-3, HD39-1, HD40-1, HD46-1, HD47-1, HD59-1, HD66-1, HD66-6, HD75-1, HD99-1, HD99-4, HD104-2). High-molecular weight DNA was extracted with the QIAamp Mini Kit (Qiagen, Hilden, Germany). Concentration was assessed on a Qubit fluorometer (Thermo Fisher Scientific, Waltham, MD, USA) and purity was assessed on a NanoDrop photometer (Thermo Fisher Scientific, Waltham, MD, USA). Native barcoding for multiplexing and PCR free ligation based ONT library preparation was performed according to manufactures protocol (Oxford Nanopore Technologies, Oxford, UK). Sequencing was done on ONT GridIOn X5 R9.4.1 flowcells. Base-calling was done with guppy version version 3.3.0 (https://community.nanoporetech.com, Oxford Nanopore Technologies, Oxford, UK). A hybrid assembly of the Illumina short-read sequencing data and Nanopore data was conducted with Unicycler version 0.4.7 [104]. All genomes with long- and short read data were successfully closed and submitted to NCBI Genbank (S3 Table for Accession numbers). Annotation of all genomes was conducted with the NCBI Prokaryotic Genome Annotation Pipeline (PGAP) [105] upon submission to Genbank.

### Analysis of intra-infection genetic diversification

One pan-genome per infection was generated by merging all genes in the presence/absence matrix produced by the Roary pipeline version 3.11.2 [106], and sequences from all single isolates from that same infection were aligned for SNP calling using the Freebayes tool [107]. Annotations of SNPs were conducted by snpEff [108] based on the annotation of the pan-genome that was carried out with GAMOLA2[109].

### Genotyping

MLST was determined by submitting contigs to pubMLST (pubmlst.org, The Department of Zoology, University of Oxford, UK) and MLST-based trees were visualized with goeBURST [110] (S2 Fig). Agr-typing was performed by comparing reference sequences for *agrD* type I, II and III (WP_001830021.1, WP_002447813.1, WP_002468352.1, respectively) using the blast algorithm against the isolates from this study (CLC Workbench, Qiagen, Hilden, Germany). We screened for CRISPR-Cas by searching for *cas1*, *cas2* and *cas10*, and if positive, the *cas* locus was typed as described previously [111]. CRISPR regions were identified with CRISPRFinder [112]. The combination of a complete *cas*-gene locus and confirmed CRISPR-region are annotated as putative functional CRISPR-Cas system.

### Analysis of phages

Prophage regions were identified and annotated with PHASTER [49]. SCC*mec* elements were identified with SCC*mec*Finder [113].

MMSeqs2 was used to taxonomically assign phage sequences using UniprotKB, nt and nr databases (most recent versions as of 14th of July 2020) [114–116]. MMSeqs2 was additionally used to annotate the phage sequences with protein domain information from PFAM (version 33.1) [117].

### Phylogenetic analysis of ST2 isolates

The evolutionary history of INF isolates of ST2 was inferred using the Maximum Likelihood method based on the General Time Reversible model. Evolutionary analyses were conducted with RaxML-NG [118,119]. The tree with the highest log-likelihood is shown (S3 Fig). A bootstrap of 1000 replicates was performed, and values above 70 are shown[120]. The initial tree for the heuristic search was randomly generated. A discrete Gamma distribution was used to model evolutionary rate differences among sites with four categories. The tree is drawn to scale, with branch lengths measured in the number of substitutions per site. The analysis based on a ’Mugsy’ alignment [121]. All positions containing gaps and missing data were eliminated. There were a total of 2288372 positions in the final dataset. Isolates BPH0662 (GCA_900086615.2) and IRL01 (GCA_009685135.1) were included as references for the two major ST2 lineages [15].

### Gene association study

The association of genes from the accessory genome to infection or commensalism was analysed. To that end a gene presence/absence matrix for the accessory genome was built with Roary version 3.11.2 running with the default parameter setting (sequence identity  =  95%) for protein alignment [106]. Each gene’s association was tested with Scoary version 1.6.16 with default parameter settings [122]. Followed by adjustment for multiple testing with the Benjamini-Hochberg[63]. Associated ORFs were annotated by hand according to gene category (main categories mobilome/chromosomal, mobilome divided into plasmid-association, phage-association, SCCmec, ACME, general mobilome (IS elements, transposases)).

### K-mer based genome wide association study

All non-clonal isolates were selected for analysis, whereas only a single isolate was randomly selected from each infection sample. In total 62 non-clonal isolates (non-clonal group) and 23 infection isolates (infection group) were analysed.

Contigs were split into overlapping 30-mers using Jellyfish (version 2.2.10, parameters used: -m 30 -s 150M -C) [123]. The k-mer sequences were saved in FASTA format and their abundances were compiled in a table.

A 2 x 2 contingency table was generated for each k-mer. To test for significant differences between the two groups, Fisher’s exact test was applied[38]. Only k-mers occurring with significantly (p-value ≤ 0.001) different abundances between both groups were considered for further analysis.

For each isolate a binary vector, indicating the presence of each k-mer, was constructed. Based on these vectors, a random forest model was trained for classifying whether an isolate belongs to the infection group or non-clonal group. The Python package Scikit-learn [124] was used for this task. Finally, k-mers, were sorted by their feature importance. Only the k-mers with a feature importance greater than zero were kept.

A pan-genome consisting of all isolates was constructed using Roary 3.12.0 [106] with results previously generated by Prokka [125]. The python packages biopython and regex were then used associate k-mers with the genomic loci they originated from.

### RNA-Seq

Five patients with INF isolates and CloNo isolates (nose isolates PFGE-identical to infection isolates) were selected (HD04, HD21, HD26, HD29, HD33)(Total number of RNA-Seq experiments n = 120). Randomly two INF isolates and two CloNo isolates were selected and grown in TSB overnight at 37°C under vigorous shaking in ambient air. Cultures were then diluted in TSB or 50% heat-inactivated, pooled human serum from 18 healthy individuals plus 50% PBS 1:500 and grown for 6h under the same conditions as before. Bacterial cells were then harvested, washed in PBS and immersed in RNAprotect (Qiagen, Hilden, Germany). Cells were pelleted after brief incubation. Cells were mechanically lysed with zirconia beads 3x20s on a tissue homogenizer (Precellys 12, Bertin, Montigny-le-Bretonneux, France) and RNA was extracted with the RNeasy mini kit (Qiagen, Hilden, Germany). RNA was quantified on a Qubit fluorometer (Thermo Fisher Scientific, Waltham, MD, USA). DNA was digested with DNA-free kit (Invitrogen, Karlsbad, CA, USA). Quality was assessed on a Bioanalyzer with RNA Nano kit (Agilent, Santa Clara, CA, USA) and only samples with a RIN >7.0 were accepted. rRNA depletion and strand specific cDNA sequencing on a HiSeq instrument (Illumina) were conducted at BGI genomics (Shenzhen, China). A mean of 11,061,832 paired-end, 150 nt Illumina reads were generated. Bases less than Q30 and adapter sequences of the reads were trimmed, and any reads shorter than 35 nt were removed using Trimmomatic version 0.36 [102]. Trimmed high-quality reads were then aligned to the assembly of isolate HD04-1 (CP052985, CP052986, and CP052987) with HISAT2 (version 2.1.0) [126]. Normalization and differential expression analyses were performed with DESeq2 [127]. The COG annotation was carried out with GAMOLA2 [109]. Volcano plots were graphed in R with the EnhancedVolcano package version 1.6.0 [128].

## Supporting information

S1 Table Patient characteristics. All but one patient were treated with a one-stage exchange of the prosthesis and did not receive antibiotic treatment 6 weeks prior to surgery and sampling. a Early: 0–3 months; delayed: >3–12 months, late: >12 months [37]. b according to[129]. c Factor: signal to cut-off, nd: not determined.
(DOCX)

S2 Table Inflammatory markers in blood and synovial fluid of patients. (DOCX)

S3 Table Number of collected isolates per patient. (DOCX)

S4 Table Isolate typing. (XLSX)

S5 Table Antibiotic susceptibility in *S. epidermidis* infection and non-clonal nose isolates. a according to EUCAST breakpoints (version 10.0).
(DOCX)

S6 Table GWAS. (XLSX)

S7 Table SNP counts within infections (INF isolates). (DOCX)

S8 Table SNP counts within nose isolates (CloNo isolates) na: not applicable. (DOCX)

S9 Table SNP counts between INF and CloNo isolates. (DOCX)

S10 Table Rearrangements between INF and CloNo isolates. (DOCX)

S11 Table Intra-clonal AckA variants. ^1^variant calling according to reference strains ATCC 12228 (Accession number CP022247 to CP022252) and RP62A (Accession number CP000029)
(DOCX)

S12 Table Regulation of *agr*-genes and *psm*-genes in RNA-Seq analysis. a all adjusted p-values ≤0.05.
(DOCX)

S1 Fig Detailed view of phenotypes in INF and nCloNo isolates. **(A)** Biofilm formation as determined by gentian violet dye microtiter plate assay of mature biofilms. **(B)** Detailed view of hemolysis of goat erythrocytes by 24h-culture supernatant **(C)** 22h growth curves in TSB as quantified by area under the curve (AUC), Growth curves for 22h at 37°C, **(D)** SNM3.**(E)** RPMI, **(F)** 70% human serum diluted with PBS.
(TIF)

S2 Fig goeBURST DLV analysis of MLST distribution in INF and nCloNo isolates[110]. Font size indicates the number of isolates per MLST. Light green indicates probable group founders, and dark green probable sub-group founders. Common nodes are displayed in light blue. Single and double locus variants are connected by lines. Circles indicate presence of INF (red) and nCloNo (grey) isolates in the respective MLST. Clonal Complex 2 Cluster 1 (CC2-I) and Cluster 2 (CC2-II) are marked in black [13].
(TIF)

S3 Fig Molecular Phylogenetic analysis of infection isolates belonging to ST2 by Maximum Likelihood method and two representative isolates of the two major ST2 lineages (BPH0662, representative of “BPH0662 clones”-lineage and IRL01, representative of “ST2-mixed” lineage[15]. (TIF)

S4 Fig Alignment of infection-associated genes to pan-genomes. **(A)** ST5 from innermost circle to outermost: HD04 (purple), HD26 (lime), HD27 (pink), HD29 (light blue), HD59 (dark blue) and significantly associated genes in the gene-based GWAS (turquoise). GC-content (black), GC-skew (+) green, GC-skew (-) purple. **(B)** all other non-ST2 STs from innermost circle to outermost: HD15 (ST212, purple), HD17 (ST290, lime), HD33 (ST87, pink), HD39 (ST297, light blue), HD40 (ST54, dark blue), HD43 (ST23, blue), HD66 (ST87, yellow), HD69 (ST130, green), HD75 (ST984, mint), HD104 (ST846, red) and significantly associated genes in the gene-based GWAS (red). GC-content (black), GC-skew (+) green, GC-skew (-) purple.
(TIF)

S5 Fig **Manhattan plots** of infection- (red) and commensalism-associated k-mers (black). Y-axis shows log -10-tranformed p-values, x-axis shows genes that k-mers map to. Dot size signifies the number of samples the k-mer is present in and colour signifies the fraction of infection and nose strains the k-mer is found in (Light red: only infection isolates, black: only in commensal isolates **(A)** Manhattan plot of k-mers mapping to the core genome (defined as present in > = 70% of samples, 2060 genes in total) **(B)** Manhattan plot of the accessory genome (gene present in at least one sample, 4581 genes in total)
(TIF)

S6 Fig Distribution of growth curve areas under the curve (AUC) in invasive and commensal *S. epidermidis* populations. AUCs (y-axis), sorted by patient (x-axis). Values were log transformed to attain symmetric distribution (indicated on y-axis label) where appropriate. Each dot represents one isolate. INF isolates are coloured in red, CloNo isolates are coloured in black. Plotted dots are means of biological duplicates. **(A)** Tryptic soy broth (TSB). **(B)** Synthetic nose media (SNM3). **(C)** 70% pooled heat-inactivated human serum with PBS. **(D)** RPMI cell culture media.
(TIF)

S7 Fig MICs (y-axis) as determined by Etest sorted by patients (x-axis). Red dots above each patient indicate individual isolates from the infection, black dots indicate nasal isolates identical to the infection clones. Dotted lines indicate susceptibility breakpoints according to EUCAST. Cases marked with a white arrow indicate likely differences in expression of resistance genes that led to divergent susceptibility test results in different clones from one and the same infection. Red arrow indicate mutations in subsets of isolates within one infection that lead to changes in susceptibility. **(A)** penicillin, **(B)**rifampicin, **(C)** daptomycin, **(D)** gentamicin and **(E)** vancomycin.
(TIF)

S8 Fig **Phenotype of colonies with IS insertion in *agrC*** (red) and with wild-type *agrC* (green). Columns represent individual patients. **(A)** Biofilm formation. **(B)** hemolysis of goat erythrocytes by culture supernatant.
(TIF)

S9 Fig Volcano plot of RNA-Seq INF isolates (n = 2 INF isolates per patient, experimental triplicates) versus CloNo isolates (n = 2 CloNo isolates per patient, experimental triplicates) after 6h of growth. Red dots signify genes with log2-fold-changes ≥2, and a Benjamini-Hochberg procedure adjusted p-value ≥0.05. Blue dots are genes with adjusted p-value ≥0.05, green dots are genes with log2-fold-changes ≥2. Genes represented by grey dots fulfil neither condition. **(A)** patient HD04, TSB; **(B)** patient HD04, 50% human serum; **(C)** patient HD21, TSB; **(D)** patient HD21, 50% human serum; **(E)** patient HD26, TSB; **(F)** patient HD26, 50% human serum; **(G)** patient HD29, TSB; **(H)** patient HD29, 50% human serum; **(I)** patient HD33, TSB; **(J)** patient HD33, 50% human serum.
(TIF)
