# Supplementary material for: Distinct clonal lineages and within-host diversification shape invasive Staphylococcus epidermidis populations
Source: PLoS Pathog. 2021 Feb 5;17(2):e1009304. doi: 10.1371/journal.ppat.1009304 (PMC7891712; doi:10.1371/journal.ppat.1009304)
Supplement: S1 Table — All but one patient were treated with a one-stage exchange of the prosthesis and did not receive antibiotic treatment 6 weeks prior to surgery and sampling. a Early: 0–3 months; delayed: >3–12 months, late: >12 months [37]. b according to[129]. c Factor: signal to cut-off, nd: not determined. (DOCX) [file ppat.1009304.s001.docx]

**Table S1: Patient characteristics**

| Patient | | PJI site | Classification  onset^a^ | | Duration of symptoms [months] | Intra-operative histopapthology  [SLIM consensus grading^b^] | Leucocyte count synovial fluid [cells/µL] | α-defensin  synovial fluid^c^  [factor] |
| --- | --- | --- | --- | --- | --- | --- | --- | --- |
| HD04 | m, 75 | hip | | early | 1 | nd | 10,940 | nd |
| HD05 | m, 81 | hip | | late | 5 | nd | 20,999 | 7.4 |
| HD12 | f, 67 | hip | | late | 3 | SLIM II | 45,357 | nd |
| HD15 | f, 60 | hip | | late | 11 | SLIM II | nd | nd |
| HD17 | m, 78 | hip | | delayed | 5 | SLIM II | nd | 5.0 |
| HD21 | f, 71 | hip | | early | 6 | SLIM II | nd | nd |
| HD25 | m, 80 | hip | | late | 8 | nd | nd | nd |
| HD26 | m, 66 | hip | | early | 8 | nd | nd | 8.9 |
| HD27 | f, 69 | hip | | early | 2 | nd | nd | nd |
| HD29 | m, 73 | hip | | early | 5 | nd | 6,508 | nd |
| HD31 | m, 64 | hip | | early | 2 | nd | nd | nd |
| HD33 | m, 53 | hip | | late | 6 | SLIM II | nd | 5.1 |
| HD39 | m, 72 | knee | | late | 9 | SLIM II | 30,870 | 4.5 |
| HD40 | f, 75 | hip | | late | 1 | nd | 74,379 | 3.7 |
| HD43 | f, 73 | hip | | delayed | 9 | SLIM II | nd | 8.1 |
| HD46 | m, 62 | knee | | late | 5 | perispacer reaction | 7,948 | 0.8 |
| HD47 | m, 53 | hip | | late | 9 | SLIM II | 236,705 | 12.0 |
| HD59 | m, 52 | hip | | early | 2 | SLIM II | nd | 3.0 |
| HD66 | f, 78 | hip | | early | 20 | SLIM II | 3,488 | nd |
| HD69 | m, 71 | hip | | early | 15 | SLIM II | nd | 7.3 |
| HD75 | m, 62 | knee | | early | 27 | nd | 25,502 | 2.8 |
| HD99 | f, 84 | knee | | late | 46 | SLIM II | 31,450 | 5.8 |
| HD104 | m, 76 | hip | | early | 11 | nd | 84,170 | 1.8 |
|  |  |  | |  |  |  |  |  |
