# Supplementary material for: Distinct clonal lineages and within-host diversification shape invasive Staphylococcus epidermidis populations
Source: PLoS Pathog. 2021 Feb 5;17(2):e1009304. doi: 10.1371/journal.ppat.1009304 (PMC7891712; doi:10.1371/journal.ppat.1009304)
Supplement: S2 Table — (DOCX) [file ppat.1009304.s002.docx]

**Table S2: Inflammatory markers in blood and synovial fluid of patients**

| Inflammatory marker | mean | range |
| --- | --- | --- |
| Blood |  |  |
| CRP [mg/dL] | 36 | 4.4-98.2 |
| Leucocyte count [per µl] (23/23) | 8.0 | 5.3-14.5 |
| PMN count [per µl] (23/23) | 5.5 | 3.17-10.2 |
| Haemoglobin [blood, g/dL] (23/23) | 11.9 | 8.7-15.3 |
| Synovia |  |  |
| Leucocyte count [per µl] (12/23) | 48.193 | 3,488-23,6705 |
| %-PMN (10/23) | 90.3 | 82-99 |
| α-defensin [quotient] (14/23) | 5.4 | 0.8-12.0 |
