# Supplementary material for: Distinct clonal lineages and within-host diversification shape invasive Staphylococcus epidermidis populations
Source: PLoS Pathog. 2021 Feb 5;17(2):e1009304. doi: 10.1371/journal.ppat.1009304 (PMC7891712; doi:10.1371/journal.ppat.1009304)
Supplement: S3 Table — (DOCX) [file ppat.1009304.s003.docx]

**S3 Table: Number of collected isolates per patient**

| Patient | # INF isolates | # CloNo isolates | # nCloNo isolates |
| --- | --- | --- | --- |
| HD04 | 10 | 9 | 3 |
| HD05 | 10 | 0 | 0 |
| HD12 | 10 | 0 | 3 |
| HD15 | 9 | 0 | 5 |
| HD17 | 9 | 0 | 1 |
| HD21 | 10 | 7 | 2 |
| HD25 | 12 | 0 | 4 |
| HD26 | 10 | 3 | 2 |
| HD27 | 2 | 1 | 1 |
| HD29 | 10 | 5 | 0 |
| HD31 | 5 | 0 | 2 |
| HD33 | 10 | 2 | 6 |
| HD39 | 10 | 0 | 3 |
| HD40 | 4 | 0 | 2 |
| HD43 | 10 | 0 | 6 |
| HD46 | 10 | 0 | 1 |
| HD47 | 5 | 0 | 3 |
| HD59 | 10 | 1 | 3 |
| HD66 | 10 | 0 | 4 |
| HD69 | 4 | 0 | 4 |
| HD75 | 10 | 0 | 4 |
| HD99 | 4 | 0 | 2 |
| HD104 | 10 | 0 | 2 |
