# Supplementary material for: Distinct clonal lineages and within-host diversification shape invasive Staphylococcus epidermidis populations
Source: PLoS Pathog. 2021 Feb 5;17(2):e1009304. doi: 10.1371/journal.ppat.1009304 (PMC7891712; doi:10.1371/journal.ppat.1009304)
Supplement: S5 Table — a according to EUCAST breakpoints (version 10.0). (DOCX) [file ppat.1009304.s005.docx]

**S5 Table: Antibiotic susceptibility in *S. epidermidis* infection and non-clonal nose isolates**

|  | Infection isolates  (n resistant ^a^) | Non-clonal nose isolates  (n resistant ^a^) | p-value |
| --- | --- | --- | --- |
| Oxacillin | 17/23 | 11/62 | <0.001 |
| Clindamycin | 10/23 | 15/62 | ns |
| Erythromycin | 14/23 | 25/62 | ns |
| Linezolid | 0/23 | 3/62 | ns |
| Gentamicin | 13/23 | 6/62 | <0.001 |
| Rifampicin | 5/23 | 0/62 | 0.001 |
| Tetracycline | 8/23 | 17/62 | ns |
| Tigecycline | 0/23 | 0/62 | ns |
| Ciprofloxacin | 19/23 | 10/62 | <0.001 |
| Cotrimoxazol | 10/23 | 2/62 | <0.001 |
| Fusidic acid | 8/23 | 11/62 | ns |
| Fosfomycin | 5/23 | 1/62 | 0.001 |
| Vancomycin | 0/23 | 0/62 | ns |
