# Supplementary material for: Distinct clonal lineages and within-host diversification shape invasive Staphylococcus epidermidis populations
Source: PLoS Pathog. 2021 Feb 5;17(2):e1009304. doi: 10.1371/journal.ppat.1009304 (PMC7891712; doi:10.1371/journal.ppat.1009304)
Supplement: S7 Table — (DOCX) [file ppat.1009304.s007.docx]

**S7 Table: SNP counts within infections (INF isolates)**

| patient | number of isolates | Total number of SNPs | non-synonymous SNPs | synonymous SNPs |
| --- | --- | --- | --- | --- |
| HD04 | 10 | 2 | 2 | 0 |
| HD05 | 10 | 45 | 38 | 6 |
| HD12 | 10 | 29 | 24 | 5 |
| HD15 | 9 | 67 | 49 | 18 |
| HD17 | 10 | 6 | 3 | 3 |
| HD21 | 10 | 4 | 4 | 0 |
| HD25 | 12 | 12 | 7 | 5 |
| HD26 | 10 | 14 | 13 | 1 |
| HD27 | 2 | 43 | 11 | 32 |
| HD29 | 10 | 8 | 6 | 2 |
| HD31 | 5 | 1 | 1 | 0 |
| HD33 | 10 | 8 | 7 | 1 |
| HD39 | 10 | 29 | 21 | 8 |
| HD40 | 5 | 0 | 0 | 0 |
| HD43 | 10 | 12 | 12 | 0 |
| HD46 | 10 | 5 | 5 | 0 |
| HD47 | 5 | 0 | 0 | 0 |
| HD59 | 10 | 2 | 2 | 0 |
| HD66 | 10 | 24 | 16 | 8 |
| HD69 | 4 | 5 | 4 | 1 |
| HD75 | 10 | 16 | 13 | 3 |
| HD99 | 4 | 5 | 3 | 2 |
