# Supplementary material for: Distinct clonal lineages and within-host diversification shape invasive Staphylococcus epidermidis populations
Source: PLoS Pathog. 2021 Feb 5;17(2):e1009304. doi: 10.1371/journal.ppat.1009304 (PMC7891712; doi:10.1371/journal.ppat.1009304)
Supplement: S8 Table — (DOCX) [file ppat.1009304.s008.docx]

**S8 Table: SNP counts within nose isolates** (CloNo isolates)

| patient | number of isolates | Total number of SNPs | non-synonymous SNPs | synonymous SNPs |
| --- | --- | --- | --- | --- |
| HD04 | 9 | 28 | 20 | 8 |
| HD21 | 7 | 7 | 3 | 4 |
| HD26 | 3 | 8 | 4 | 4 |
| HD27 | 1 | na | na | na |
| HD29 | 7 | 0 | 0 | 0 |
| HD33 | 2 | 7 | 5 | 2 |
| HD59 | 1 | na | na | na |
