# Supplementary material for: Distinct clonal lineages and within-host diversification shape invasive Staphylococcus epidermidis populations
Source: PLoS Pathog. 2021 Feb 5;17(2):e1009304. doi: 10.1371/journal.ppat.1009304 (PMC7891712; doi:10.1371/journal.ppat.1009304)
Supplement: S9 Table — (DOCX) [file ppat.1009304.s009.docx]

**S9 Table: SNP counts between INF and CloNo isolates**

|  | Total number of SNPs | non-synonymous SNPs | synonymous SNPs | dN/dS |
| --- | --- | --- | --- | --- |
| HD04 | 0 | 0 | 0 | + recombination |
| HD21 | 102 | 72 | 30 | + recombination |
| HD26 | 0 | 0 | 0 |  |
| HD27 | 14 | 5 | 9 | + recombination |
| HD29 | 150 | 110 | 40 | + recombination |
| HD33 | 130 | 56 | 64 | + recombination |
| HD59 | 98 | 66 | 32 | + recombination |
