# Supplementary material for: Distinct clonal lineages and within-host diversification shape invasive Staphylococcus epidermidis populations
Source: PLoS Pathog. 2021 Feb 5;17(2):e1009304. doi: 10.1371/journal.ppat.1009304 (PMC7891712; doi:10.1371/journal.ppat.1009304)
Supplement: S10 Table — (DOCX) [file ppat.1009304.s010.docx]

**S10 Table: Rearrangements between INF and CloNo isolates**

| Patient | Locus of genetic rearrangement between PFGE identical nose and infection isolates |
| --- | --- |
| HD04 | putative phage associated amidase (NCTC13924_02390) |
| HD21 | deletion of *ccrAB4* in INF |
| HD26 | none |
| HD27 | SCC*mec* fragment containing *ccrC*, *hsdRSM* deleted in CloNo isolate |
| HD29 | deletion of CRISPR-Cas containing ACME fragment (deleted in INF)  recombinations in plasmid (recombinase (*sin*), beta-lactam sensor (*blaR1*), IS3-family transposase (HH313_000048) and a plasmid replication protein (BAU36305.1)) |
| HD33 | Deletion of ACME (deleted in INF)  recombination in region corresponding to nt 90610-154417 in *S. epidermidis* NCTC13924 *(uhpT*, *opuCC_2*, *gehD*, *bioA*, *kce*, *fadB*, *pflB*, YfcC family protein, *opp-1F*, *argF*, *opuCA_2*, *opp-1C*, *lcdH_2)* |
| HD59 | deletion of CRISPR-Cas containing ACME fragment (deleted in INF) |
