# Supplementary material for: Distinct clonal lineages and within-host diversification shape invasive Staphylococcus epidermidis populations
Source: PLoS Pathog. 2021 Feb 5;17(2):e1009304. doi: 10.1371/journal.ppat.1009304 (PMC7891712; doi:10.1371/journal.ppat.1009304)
Supplement: S11 Table — 1variant calling according to reference strains ATCC 12228 (Accession number CP022247 to CP022252) and RP62A (Accession number CP000029) (DOCX) [file ppat.1009304.s011.docx]

**S11 Table: Intra-clonal AckA variants**

| Patient ID and AckA variant ID | fraction of all analysed colonies | variant^1^ |
| --- | --- | --- |
| HD15_ackA_var1 | 7 of 9 | A119T |
| HD15_ackA_var2 | 2 of 9 | E254K, A119T |
| HD33_ackA_var1 | 3 of 10 | wt |
| HD33_ackA_var2 | 5 of 10 | T239A |
| HD33_ackA_var3 | 1 of 10 | G278R |
| HD39_ackA_var1 | 5 of 10 | M154T |
| HD39_ackA_var2 | 4 of 10 | R35H |
| HD39_ackA_var3 | 1 of 10 | G345E |
| HD43_ackA_var1 | 4 of 9 | P231S, M319I |
| HD43_ackA_var2 | 3 of 9 | M319I |
| HD43_ackA_var3 | 1 of 9 | V324F, M319I |
| HD43_ackA_var4 | 1 of 9 | P246S, M319I |
| HD46_ackA_var1 | 1 of 9 | wt |
| HD46_ackA_var2 | 3 of 9 | F304L |
| HD46_ackA_var3 | 5 of 9 | N123K |
| HD75_ackA_var1 | 1 of 10 | E107D, V324I, K397Q, D409N |
| HD75_ackA_var2 | 8 of 10 | A119V, E107D, V324I, K397Q, D409N |
| HD75_ackA_var3 | 1 of 10 | F229L, E107D, V324I, K397Q, D409N |
| HD99_ackA_var1 | 1 of 4 | wt |
| HD99_ackA_var2 | 2 of 4 | R296_S297insC |
| HD99_ackA_var3 | 1 of 4 | E333K |
