# Supplementary material for: Distinct clonal lineages and within-host diversification shape invasive Staphylococcus epidermidis populations
Source: PLoS Pathog. 2021 Feb 5;17(2):e1009304. doi: 10.1371/journal.ppat.1009304 (PMC7891712; doi:10.1371/journal.ppat.1009304)
Supplement: S12 Table — a all adjusted p-values ≤0.05. (DOCX) [file ppat.1009304.s012.docx]

**S12 Table: Regulation of *agr-*genes and *psm*-genes in RNA-Seq analysis**

|  | Gene | fold-change TSB^a^ | fold-change 50% hS^a^ |
| --- | --- | --- | --- |
| HD21 |  |  |  |
|  | *agrA* | - | - |
|  | *agrB* | 1422 | 17.8 |
|  | *agrC* | - | - |
|  | *agrD* | 827 | 47.2 |
|  | *psmα* | 0.09 | - |
|  | *psmβ1a* | - | - |
|  | *psmβ1b* | - | - |
|  | *psmβ2* | 0.03 | 0.06 |
|  | *psmβ3* | 0.009 | 0.21 |
|  | *psmδ* | - | - |
|  |  |  |  |
| HD26 |  |  |  |
|  | *agrA* | - | - |
|  | *agrB* | - | - |
|  | *agrC* | - | - |
|  | *agrD* | - | 4.4 |
|  | *psmα* | - | - |
|  | *psmβ1a* | - | 25.5 |
|  | *psmβ1b* | - | 42.6 |
|  | *psmβ2* | - | 28.5 |
|  | *psmβ3* | - | 93.7 |
|  | *psmδ* | - | 19.5 |
|  |  |  |  |
| HD29 |  |  |  |
|  | *agrA* | - | - |
|  | *agrB* | - | - |
|  | *agrC* |  | - |
|  | *agrD* | - | - |
|  | *psmα* | - | - |
|  | *psmβ1a* | - | 7.5 |
|  | *psmβ1b* | 9.14 | 5.2 |
|  | *psmβ2* | - | 5.7 |
|  | *psmβ3* | - | 6.4 |
|  | *psmδ* | - | - |
|  |  |  |  |
| HD33 |  |  |  |
|  | *agrA* | 0.31 | - |
|  | *agrB* | - | - |
|  | *agrC* | - | - |
|  | *agrD* | - | - |
|  | *psmα* | 0.19 | - |
|  | *psmβ1a* | 245.9 | 29.6 |
|  | *psmβ1b* | 0.1 | - |
|  | *psmβ2* | 0.09 | - |
|  | *psmβ3* | 0.15 | - |
|  | *psmδ* | - | - |
