# Supplementary figures and images for: Distinct clonal lineages and within-host diversification shape invasive Staphylococcus epidermidis populations
Source: PLoS Pathog. 2021 Feb 5;17(2):e1009304. doi: 10.1371/journal.ppat.1009304 (PMC7891712; doi:10.1371/journal.ppat.1009304)

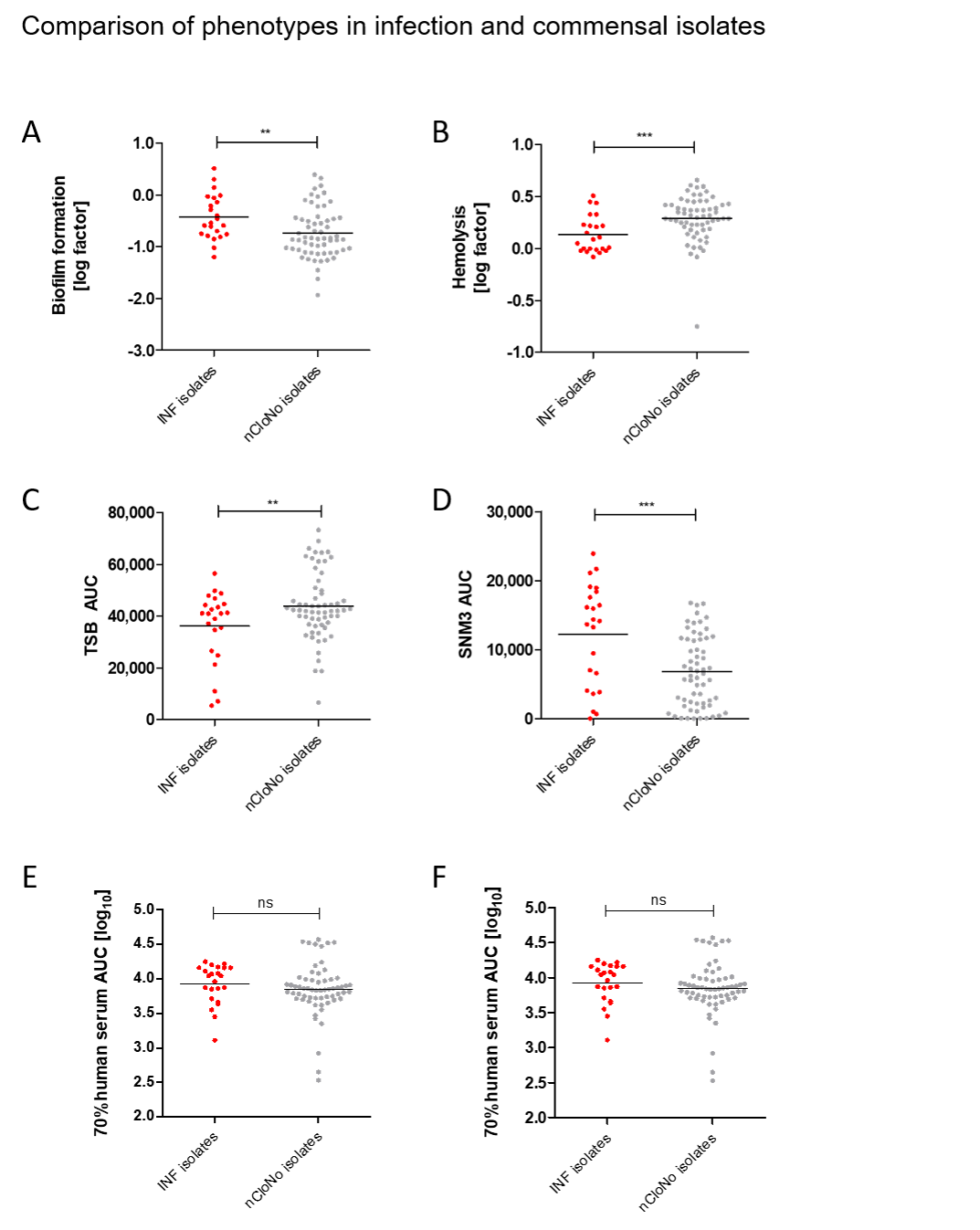

Supplement: S1 Fig — (A) Biofilm formation as determined by gentian violet dye microtiter plate assay of mature biofilms. (B) Detailed view of hemolysis of goat erythrocytes by 24h-culture supernatant (C) 22h growth curves in TSB as quantified by area under the curve (AUC), Growth curves for 22h at 37°C, (D) SNM3.(E) RPMI, (F) 70% human serum diluted with PBS. (TIF) [file ppat.1009304.s013.tif]

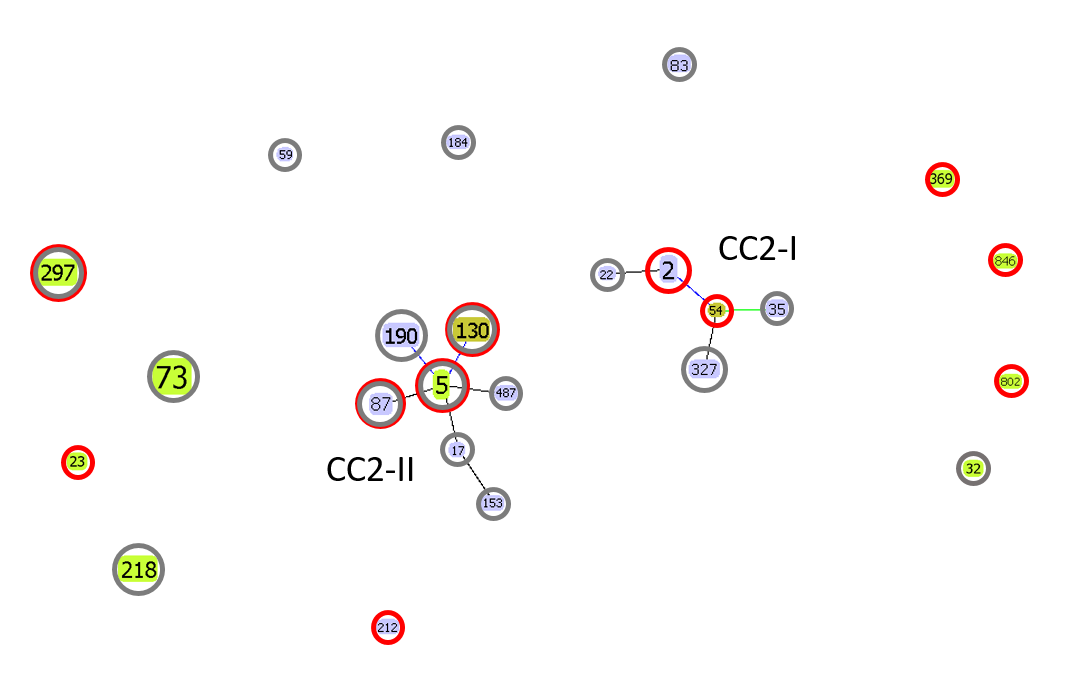

Supplement: S2 Fig — Font size indicates the number of isolates per MLST. Light green indicates probable group founders, and dark green probable sub-group founders. Common nodes are displayed in light blue. Single and double locus variants are connected by lines. Circles indicate presence of INF (red) and nCloNo (grey) isolates in the respective MLST. Clonal Complex 2 Cluster 1 (CC2-I) and Cluster 2 (CC2-II) are marked in black [13]. (TIF) [file ppat.1009304.s014.tif]

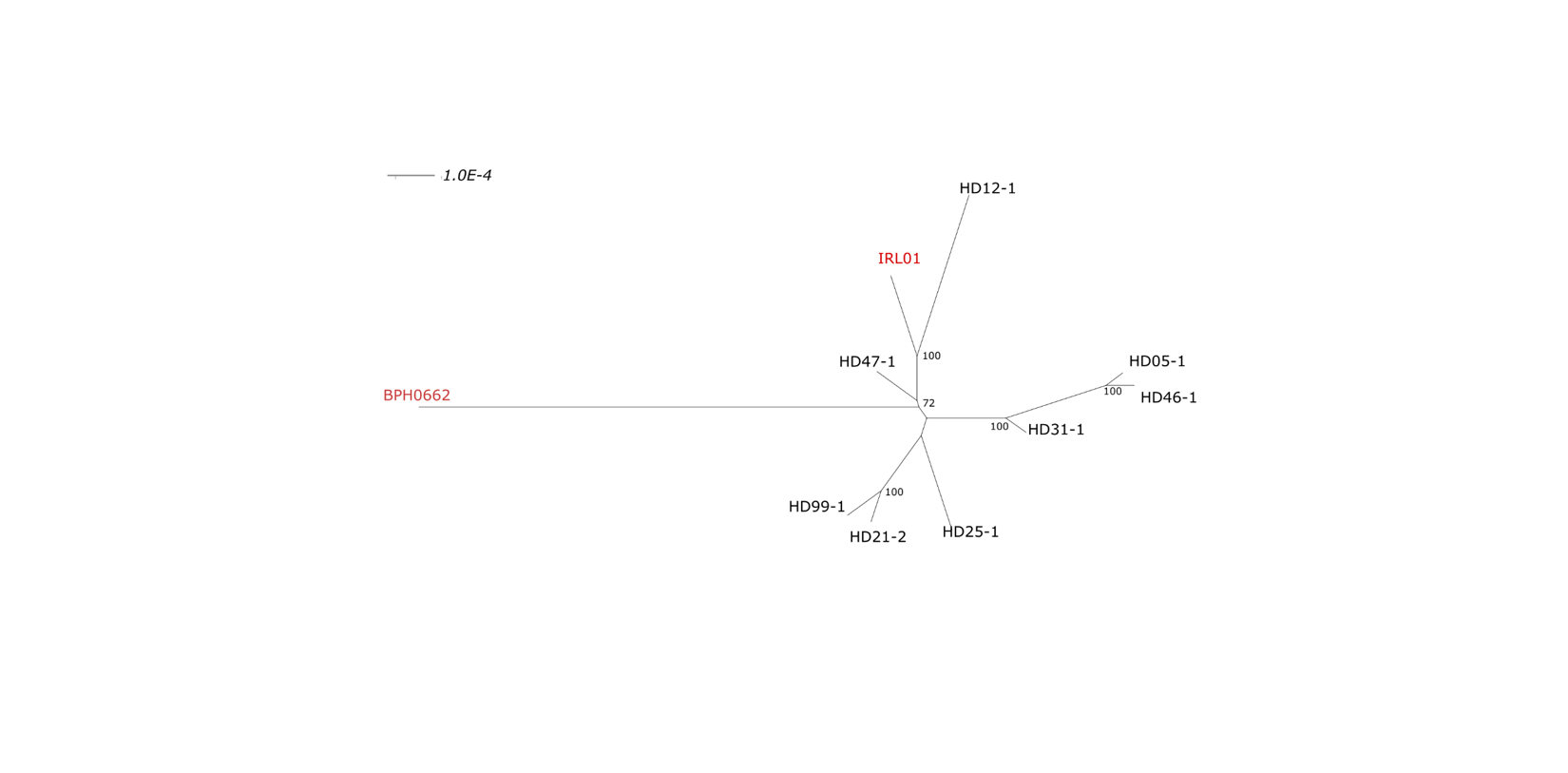

Supplement: S3 Fig — (TIF) [file ppat.1009304.s015.tif]

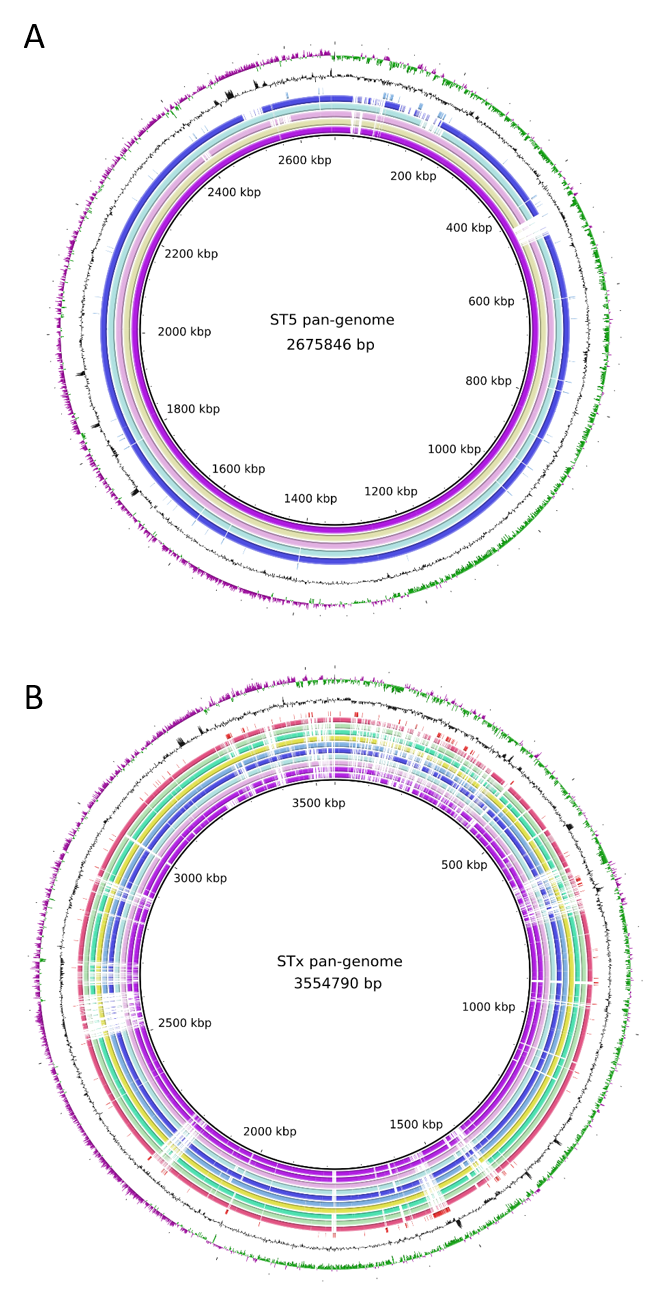

Supplement: S4 Fig — (A) ST5 from innermost circle to outermost: HD04 (purple), HD26 (lime), HD27 (pink), HD29 (light blue), HD59 (dark blue) and significantly associated genes in the gene-based GWAS (turquoise). GC-content (black), GC-skew (+) green, GC-skew (-) purple. (B) all other non-ST2 STs from innermost circle to outermost: HD15 (ST212, purple), HD17 (ST290, lime), HD33 (ST87, pink), HD39 (ST297, light blue), HD40 (ST54, dark blue), HD43 (ST23, blue), HD66 (ST87, yellow), HD69 (ST130, green), HD75 (ST984, mint), HD104 (ST846, red) and significantly associated genes in the gene-based GWAS (red). GC-content (black), GC-skew (+) green, GC-skew (-) purple. (TIF) [file ppat.1009304.s016.tif]

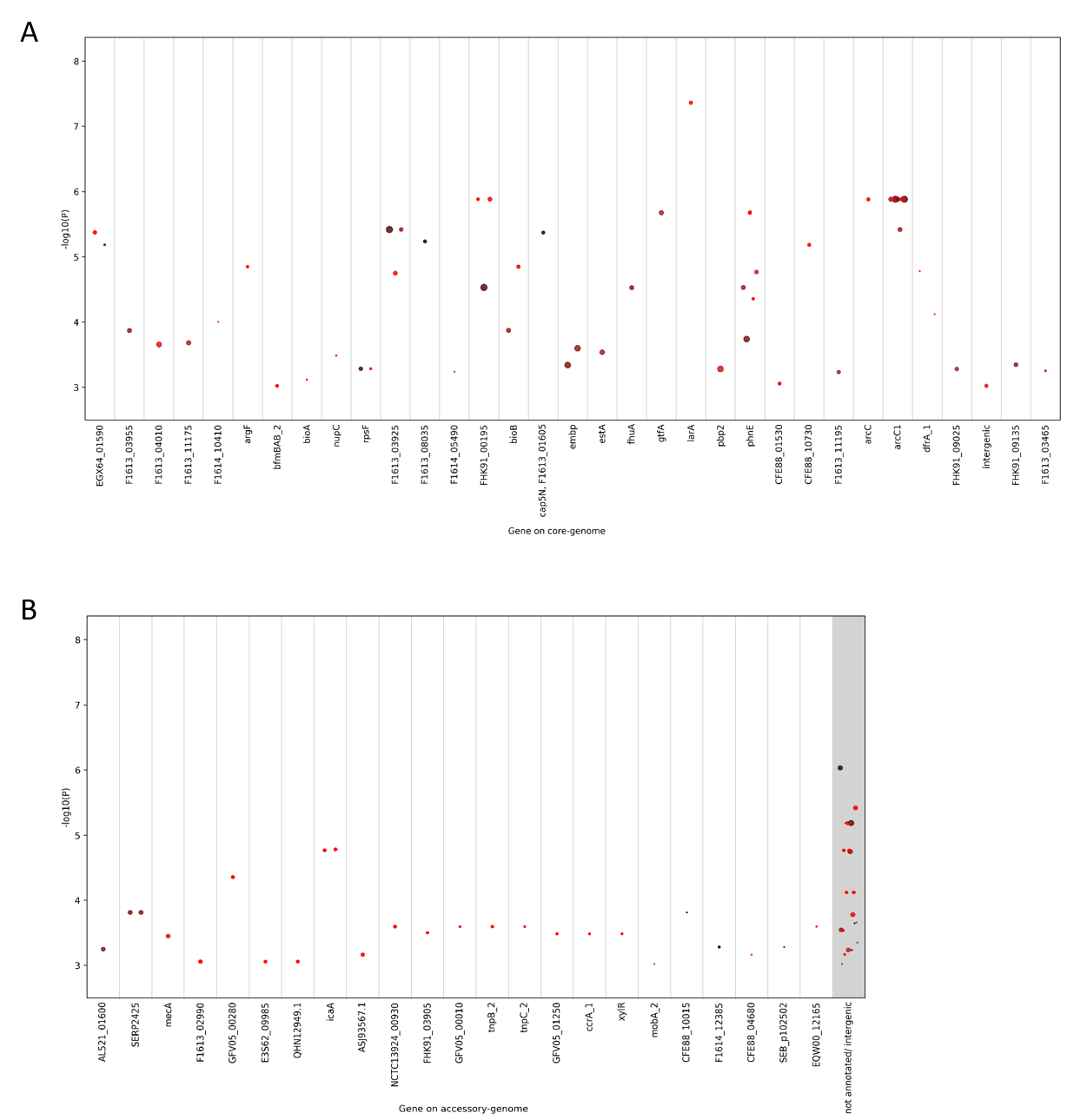

Supplement: S5 Fig — Manhattan plots of infection- (red) and commensalism-associated k-mers (black). Y-axis shows log -10-tranformed p-values, x-axis shows genes that k-mers map to. Dot size signifies the number of samples the k-mer is present in and colour signifies the fraction of infection and nose strains the k-mer is found in (Light red: only infection isolates, black: only in commensal isolates (A) Manhattan plot of k-mers mapping to the core genome (defined as present in > = 70% of samples, 2060 genes in total) (B) Manhattan plot of the accessory genome (gene present in at least one sample, 4581 genes in total) (TIF) [file ppat.1009304.s017.tif]

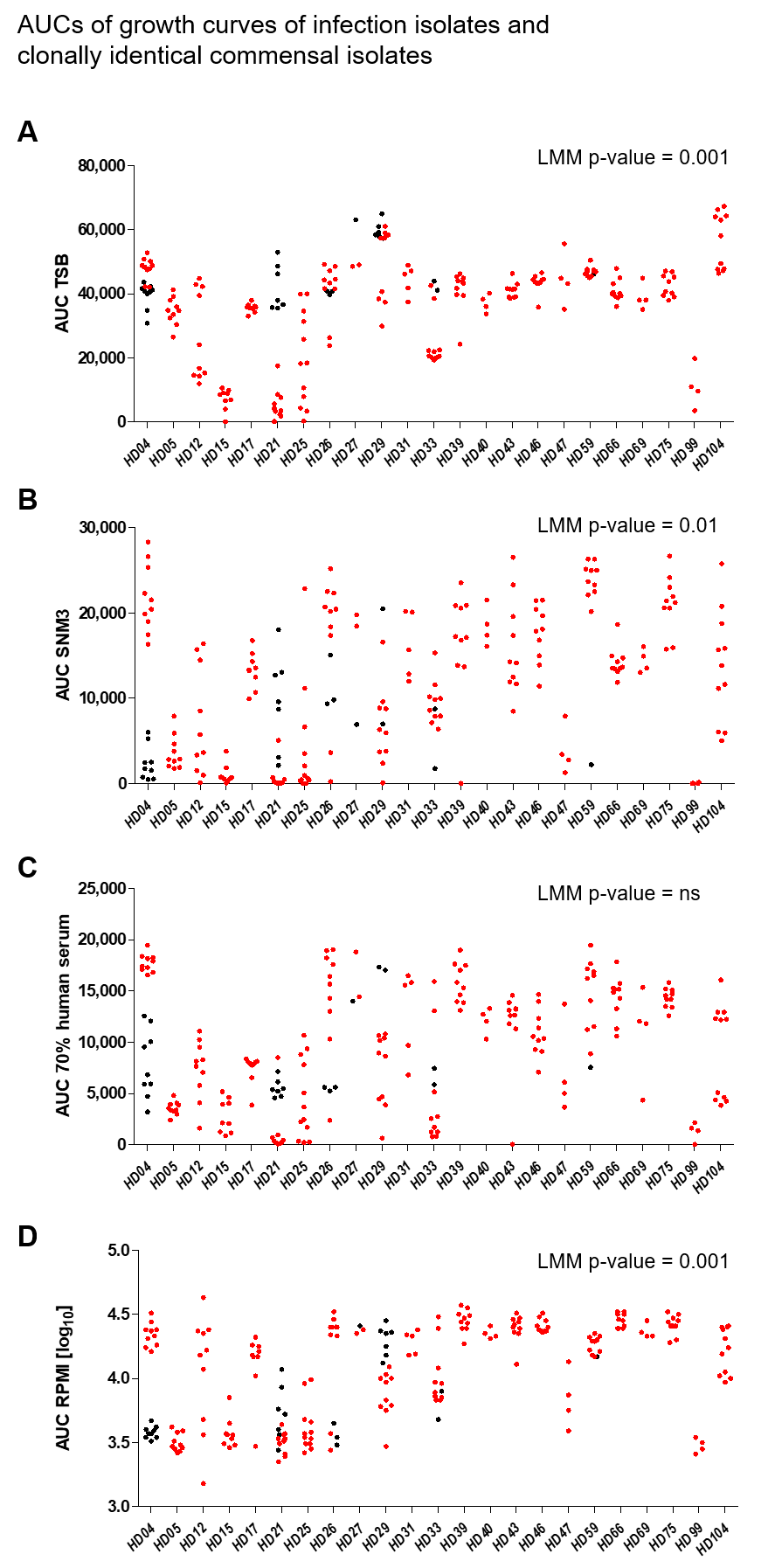

Supplement: S6 Fig — Values were log transformed to attain symmetric distribution (indicated on y-axis label) where appropriate. Each dot represents one isolate. INF isolates are coloured in red, CloNo isolates are coloured in black. Plotted dots are means of biological duplicates. (A) Tryptic soy broth (TSB). (B) Synthetic nose media (SNM3). (C) 70% pooled heat-inactivated human serum with PBS. (D) RPMI cell culture media. (TIF) [file ppat.1009304.s018.tif]

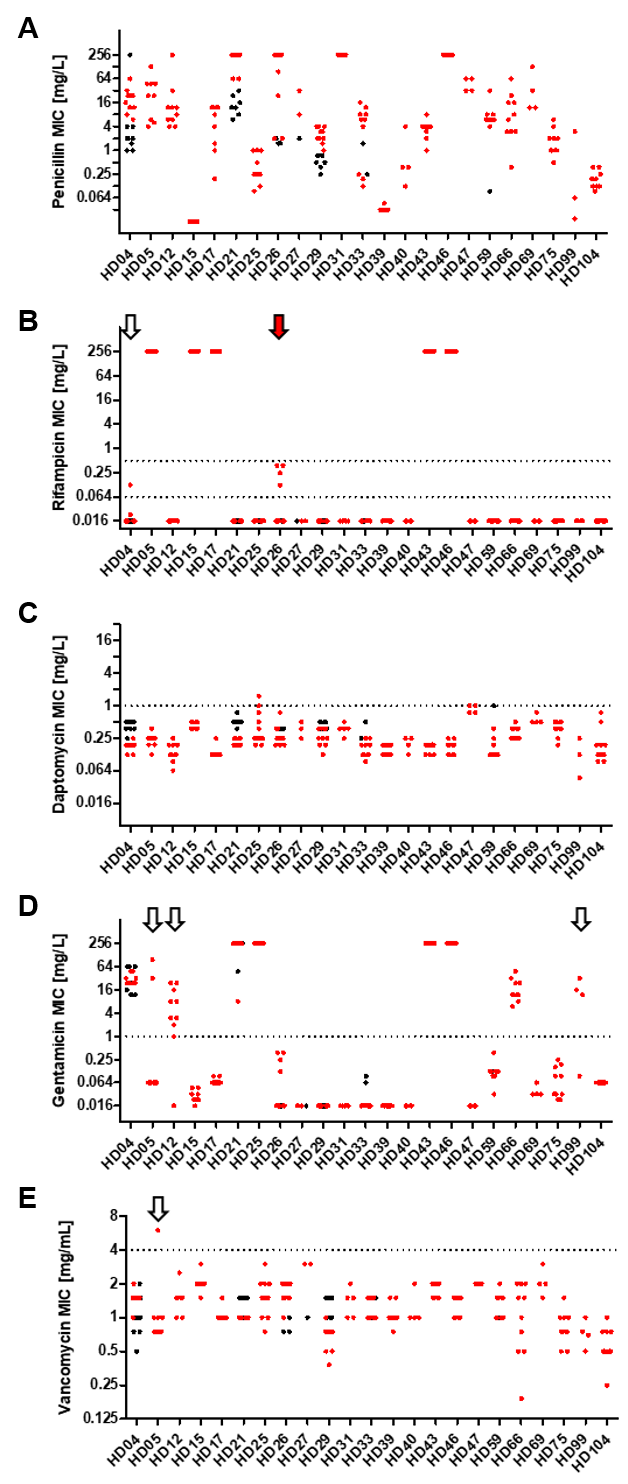

Supplement: S7 Fig — Red dots above each patient indicate individual isolates from the infection, black dots indicate nasal isolates identical to the infection clones. Dotted lines indicate susceptibility breakpoints according to EUCAST. Cases marked with a white arrow indicate likely differences in expression of resistance genes that led to divergent susceptibility test results in different clones from one and the same infection. Red arrow indicate mutations in subsets of isolates within one infection that lead to changes in susceptibility. (A) penicillin, (B)rifampicin, (C) daptomycin, (D) gentamicin and (E) vancomycin. (TIF) [file ppat.1009304.s019.tif]

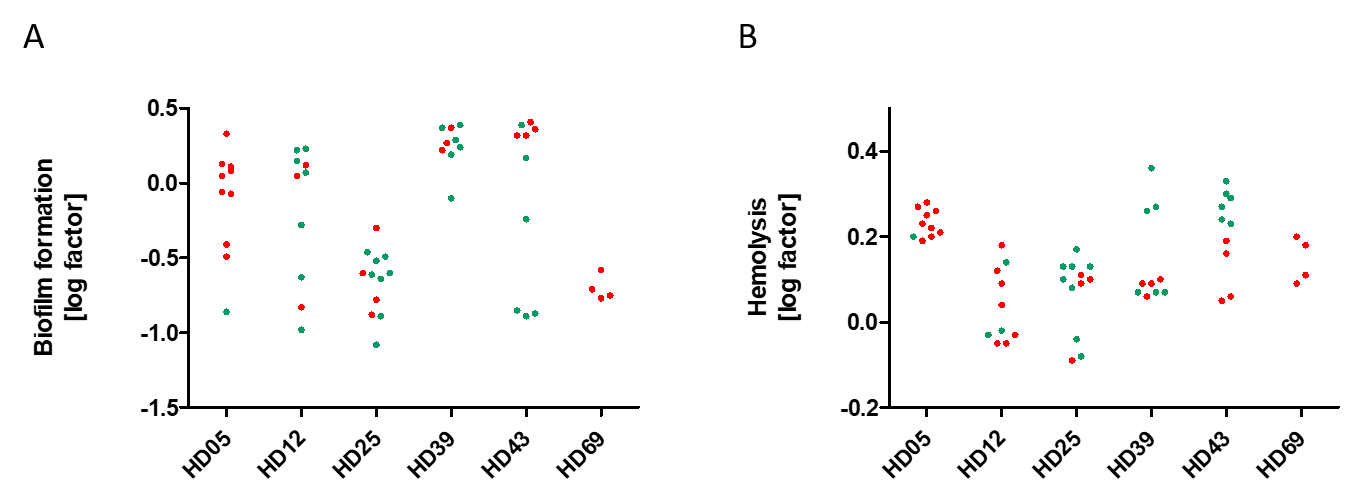

Supplement: S8 Fig — Phenotype of colonies with IS insertion in agrC (red) and with wild-type agrC (green). Columns represent individual patients. (A) Biofilm formation. (B) hemolysis of goat erythrocytes by culture supernatant. (TIF) [file ppat.1009304.s020.tif]

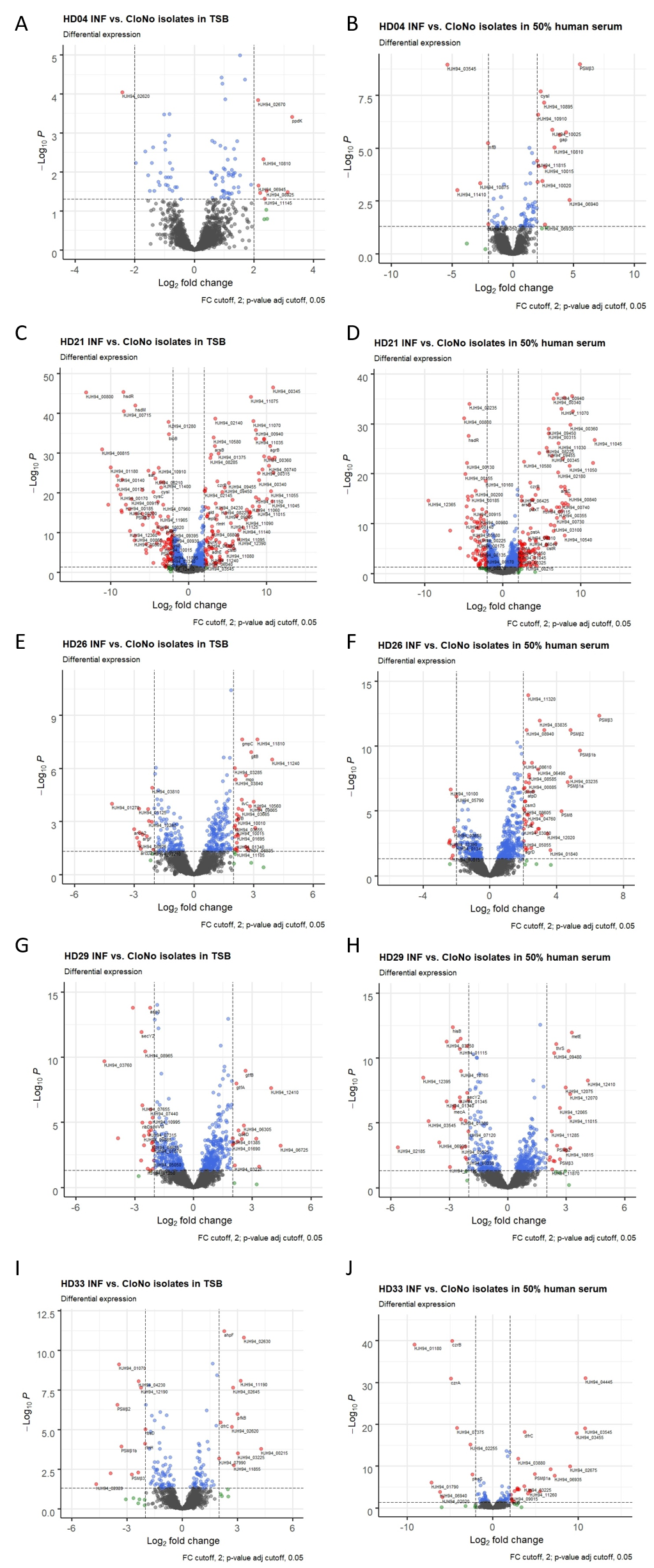

Supplement: S9 Fig — (A) patient HD04, TSB; (B) patient HD04, 50% human serum; (C) patient HD21, TSB; (D) patient HD21, 50% human serum; (E) patient HD26, TSB; (F) patient HD26, 50% human serum; (G) patient HD29, TSB; (H) patient HD29, 50% human serum; (I) patient HD33, TSB; (J) patient HD33, 50% human serum. (TIF) [file ppat.1009304.s021.tif]
